# Risk of major adverse limb events in patients with type 2 diabetes mellitus receiving sodium glucose cotransporter 2 inhibitors and glucagon-like peptide-1 receptor agonists: A population-based retrospective cohort study

**DOI:** 10.3389/fphar.2022.869804

**Published:** 2022-09-13

**Authors:** Yen-Chieh Lee, Yaa-Hui Dong, Wei-Shun Yang, Li-Chiu Wu, Jou-Wei Lin, Chia-Hsuin Chang

**Affiliations:** ^1^ Department of Family Medicine, Cathay General Hospital, Taipei, Taiwan; ^2^ Department of Medicine, College of Medicine, Fu Jen Catholic University, New Taipei City, Taiwan; ^3^ Department of Pharmacy, College of Pharmaceutical Sciences, National Yang Ming Chiao Tung University, Taipei, Taiwan; ^4^ Institute of Public Health, School of Medicine, National Yang Ming Chiao Tung University, Taipei, Taiwan; ^5^ Institute of Hospital and Health Care Administration, School of Medicine, National Yang Ming Chiao Tung University, Taipei, Taiwan; ^6^ Department of Internal Medicine, National Taiwan University Hospital Hsin-Chu Branch, Hsinchu City, Taiwan; ^7^ The Graduate Institute of Medical Genomics and Proteomics, National Taiwan University, Taipei, Taiwan; ^8^ Department of Internal Medicine, National Taiwan University Hospital, Taipei, Taiwan; ^9^ Department of Internal Medicine, National Taiwan University Hospital Yunlin Branch, Douliu City, Taiwan; ^10^ Cardiovascular Center, National Taiwan University Hospital Yunlin Branch, Douliu City, Taiwan; ^11^ Department of Medicine, College of Medicine, National Taiwan University, Taipei, Taiwan; ^12^ Institute of Epidemiology and Preventive Medicine, College of Public Health, National Taiwan University, Taipei, Taiwan

**Keywords:** sodium-glucose cotransporter-2 inhibitor, critical limb ischemia, lower extremity amputation, glucagon-like peptide-1 receptor agonist, dipeptidyl peptidase-4 inhibitor, type 2 diabetes

## Abstract

**Background:** Both sodium glucose cotransporter 2 inhibitors (SGLT-2i) and glucagon-like peptide-1 receptor agonists (GLP-1RA) have cardiovascular protective effects in patients with type 2 diabetes mellitus. However, the comparative risk of GLP-1RA versus SGLT-2i for major adverse limb events remains unknown.

**Materials and methods:** We studied a nationwide cohort involving 123,048 diabetes patients 20–100 years of age who initiated a SGLT-2i or GLP-1RA during 2012 and 2017. The patients in the two groups were matched by propensity score (PS), and incidence rates for hospitalization for major adverse limb events, critical limb ischemia (CLI) and lower extremity amputation (LEA), were assessed. Cox proportional hazards regression was applied to estimate hazard ratios (HRs) between patients receiving SGLT-2i as compared with GLP-1RA. The modification effects of age, a history of established cardiovascular disease, and chronic kidney disease were examined. In addition, use of dipeptidyl peptidase-4 inhibitor (DPP-4i) was chosen as a second active comparator.

**Results:** After PS-matching, a total of 13,378 SGLT-2i and 13,378 GLP-1RA initiators were identified. Use of SGLT-2i was not associated with an increased risk for hospitalization for CLI and LEA, either compared with GLP-1RA (HR, 1.13; 95% CI, 0.77–1.65 and 1.27; 95% CI, 0.63–2.55, respectively) or compared with DPP-4i use (HR, 1.06; 95% CI, 0.75–1.50 and HR, 0.80; 95% CI, 0.42–1.53, respectively). Although the study was underpowered to explore potential effect modification, a trend of higher risks for LEA was noted among SGLT-2i users with cardiovascular disease as compared with either GLP-1RA or DPP-4i.

**Conclusion:** Use of SGLT-2i was not associated with higher risks for hospitalization for CLI and LEA as compared with reference drugs. Further large-scale studies are needed for a precise risk estimation.

## Introduction

Critical limb ischemia (CLI) is one of the most devastating patterns of peripheral artery disease (PAD), but often an under-recognized complication for patients with diabetes mellitus. The onset of CLI is often dramatic, causing considerable morbidity and mortality, especially from subsequent management with major lower extremity amputation (LEA) (Faglia et al., 2009). With clinical and public health efforts, a decline in rate of LEA was observed in the past two decades but the case number has been rising since 2019, particularly in young and middle-aged adults (Geiss et al., 2019; Paul et al., 2021).

Novel anti-diabetic agents, sodium-glucose co-transporter 2 inhibitors (SGLT-2i) and glucagon-like peptide-1 receptor agonists (GLP-1RA), have shown promising effects on cardiovascular protection in patients with type 2 diabetes (Zinman et al., 2015; Marso et al., 2016; Neal et al., 2017; Wiviott et al., 2018; Gerstein et al., 2019). However, the potential risk of CLI or LEA associated with these agents remains an issue of great concern. In the CANagliflozin cardioVascular Assessment Study (CANVAS), the use of canagliflozin has doubled the risk for LEA among patients with established cardiovascular disease (CVD) or multiple risk factors (Neal et al., 2017). On the other hand, other evidence from clinical trials, including those involving other SGLT-2i (i.e., empagliflozin or dapagliflozin), did not show meaningfully harmful effects (Li et al., 2018; Scheen, 2018; Verma and Bhatt, 2019). Evidence from observational studies, including some comparing SGLT-2i with GLP-1RA (Chang et al., 2018; Ueda et al., 2018; Fralick et al., 2020; Hsiao et al., 2021; Patorno et al., 2021; Paul et al., 2021), was divergent and conflicting (Chang et al., 2018; Ryan et al., 2018; Ueda et al., 2018; Fralick et al., 2020; Caparrotta et al., 2021; Hsiao et al., 2021; Li et al., 2021; Patorno et al., 2021; Paul et al., 2021), probably due to heterogeneity in terms of study design and analysis, specific types of SGLT-2i, characteristics of the included populations, and the choice of comparator drugs. Given complex and multifactorial reasons that drive diabetes patients toward amputation instead of revascularization procedures, few studies had evaluated comprehensively the safety of SGLT-2i, as compared with GLP-1RA, on the composite major adverse limb outcome: CLI, which was at the late stage of the broad spectrum of PAD (Bonaca and Beckman, 2018). Moreover, based on the signal warning about the risk of using canagliflozin from the CANVAS trial, questions remain regarding to whether this is restricted to a specific drug or a class effect that also applies to other SGLT-2i (Khouri et al., 2018). Due to unavailability or limited availability of the data concerning patients’ risk factors and lack of measurements about disease severity in previous studies, potential residual confounding by these clinical parameters remains a concern and the comparability between SGLT-2i and reference drugs is still problematic.

In light of these, we conducted a retrospective cohort study based on the nationwide population-based data. With clinical laboratory data and empirically abundant information that would serve as proxies for existence or severity of important risk factors for CLI and LEA, propensity score (PS) models were built for extensive covariate control. Under the hypothesis that use of SGLT-2i was not associated with higher risks for major adverse limb events, we compared the risks of CLI and LEA among patients who initiated empagliflozin and dapagliflozin, the most widely used medications in the SGLT-2i class in Taiwan during study period, with those initiated GLP-1RA between PS matched groups. The reasons that GLP-RA was chosen as a comparator are as followed: it is also a second-line treatment for diabetes (Dong et al., 2022a); it shares similar cardioprotective property with SGLT-2i (Dong et al., 2022b); it has not been reported with an increased risk for lower limb adverse event; and evidence from previous studies has shown great similarity between SGLT-2i and GLP-1RA initiators in age and cardiovascular risk profiles, which are two crucial risk factors for lower limbs adverse events (Fralick et al., 2019; Fralick et al., 2020). In addition, we hypothesized that the risk estimates of lower limb adverse events should be robust when the comparison was made with different reference drugs. Thus, the other class of incretin-based antidiabetic agent, dipeptidyl peptidase-4 inhibitor (DPP-4i), was selected as an alternative active comparator to SGLT-2i in examining the risk for CLI and LEA.

## Materials and methods

### Data source

The data used in the current study were obtained from the Applied Health Research Data Integration Service of the National Health Insurance (NHI) Administration, Taiwan (case number: B201905310001 and B202201140002). This set of claims data included demographics, diagnoses, procedures, pharmacy, and prescription information from outpatient visits and hospital admissions of almost all population in Taiwan, and more importantly, provided anthropometric measurements and laboratory test results uploaded from medical facilities that were not previously available in NHI Research Database (NHIRD) (Lee et al., 2021). Ethical approval for this study was waived by National Taiwan University Research Ethics Committee (202003060W).

### Study population and study drugs

This dataset exclusively contained all the patients, either outpatient or hospital admission, diagnosed with diabetes mellitus (International Classification of Diseases: ICD-9-CM codes of 250 or ICD-10-CM codes of E08, E09, E10, E11, or E13). Those who began to receive SGLT-2i or GLP-1RA from the NHIRD between 1 January 2012 (when the first GLP-1RA was reimbursed by NHI) and 31 December 2017 were included as study population. During the study period, two SGLT-2i (dapagliflozin, empagliflozin) and two GLP-1RA (liraglutide, dulaglutide) were widely used in Taiwan; therefore, the present analysis focused on these four drugs (see Supplementary Table S1 for codes). The cohort entry, or the index date, was defined as the first day of study drug dispensation. To ensure a new-user design, patients who initiated a SGLT-2i or a GLP-1RA must have had a period of 2 years without any study drug prescriptions. We also excluded patients who received both GLP-1RA or SGLT-2i and those who received more than one GLP-1RA or one SGLT-2i on the cohort entry date.

We then excluded patients with type 1 diabetes, cancer, cirrhosis, or other critical conditions such as dialysis and organ transplantation (Supplementary Table S2). Those who aged less than 20 or more than 100 years were also excluded. Subsequently, the patients who did not have any health encounter, either outpatient visit or hospital admission, within 1 year before the index date, were removed from further analysis to ensure the status of continuous NHI enrollment. Then, the patients with outcome occurrence on the index date were also excluded because the temporal relation of exposure and outcome could hardly be ascertained.

### Outcomes and follow-up

Our primary outcome of interest was incident major adverse limb events, defined by the occurrence of CLI that required hospitalization for either medical or interventional treatment (revascularization or LEA) during follow-up. The occurrence of CLI was ascertained based on the existence of ICD-9/10 diagnosis or procedure codes and the NHI reimbursement codes in the inpatient claims (Supplementary Table S3). Also, in a secondary outcome definition, risks of SGLT-2i use were investigated specifically for non-traumatic LEA to facilitate comparison with previous studies.

During the follow-up, the study drug might be discontinued or changed to other medication. Drug discontinuation was defined as a more than 45-day grace period between the end of one prescription and the start of the other, while drug switch was defined as a dispensation of GLP-1RA for initiators of SGLT-2i or vice versa. According to these definitions, we applied two alternative follow-up schemes. First, in the “on-treatment” approach, the follow-up started from the index date, and ended on the date of outcome occurrence, study drug discontinuation or switch, death, or study termination (31 December 2018), whichever came earlier. Second, in the “intention-to-treat” approach aiming to capture the latent effect of the study drugs and to reduce informative censoring related to treatment discontinuation or change, the patients were followed from the index date to the date of outcome occurrence, death, or study termination.

### Covariate assessment

We defined the characteristics of the patients during the 6 months before each subject’s index. We assessed an extensive set of prespecified covariates (reflecting status on the index date) including demographic data, coexisting medical conditions, treatment with selected medications that were possibly associated with both the use of the study drugs and the risk of outcome occurrence. The use of healthcare services that potentially served as proxies for clinical disease severity and were potentially predictive of the risk of outcome including numbers of hospital admissions, numbers of outpatient visit due to cardiovascular episodes, specialty of study drug prescribers, and whether patients received cardiovascular-related laboratory test or examinations were also assessed (Supplementary Tables S4, S5 provides detailed covariate information). Systolic blood pressure and laboratory test results, including glycated hemoglobin (HbA1c), serum creatinine, low-density lipoprotein (LDL)-cholesterol, within 90–180 days before the index date were used to balance the baseline characteristics between the two comparison groups (Supplementary Table S6). CKD-EPI equation, incorporating age, sex, and serum creatinine, was used to calculate estimated glomerular filtration rate (eGFR) (Levey et al., 2009).

### Statistical analysis

We applied propensity score matching design to balance baseline characteristics between two treatment groups. We estimated baseline PS by using logistic regression models that contained all variables shown in Table 1 to predict the probability of initiating SGLT-2i. The missing-indicator method was used to handle missing information on four important clinical parameters. Instead of excluding patients with missing data, the method adds an extra category in the variable to indicate the value is missing; for example, the HbA1c values (%) were categorized as: >9.0, 7.0–9.0, <7.0, and missing. Therefore, each participant can still be included in the analysis, reducing the loss of statistical power (Choi et al., 2019).

**TABLE 1:** Baseline characteristics of study population comparing sodium-glucose cotransporter type-2 inhibitor (SGLT-2i) with glucagon-like peptide 1 receptor agonist use before and after propensity score matching.

	Before matching (*n* = 123,048)			1:1 PS-matched cohort (*n* = 26,756)		
Covariates^a^	SGLT-2i initiators (*n* = 108,920)	GLP-1RA initiators (*n* = 14,128)	Standardized difference	SGLT-2i initiators (*n* = 13,378)	GLP-1RA initiators (*n* = 13,378)	Standardized difference
Demographics						
Age in years, mean (SD)	57.15 (12.56)	54.23 (13.89)	0.220	53.65 (13.36)	54.04 (13.77)	−0.029
Men	57.25	48.80	0.170	49.47	49.19	0.006
Overweight and obesity, %	3.78	10.47	−0.262	9.75	9.72	0.001
Smoking	1.74	1.84	−0.008	1.82	1.88	−0.005
Clinical parameters						
HbA1c, %						
>9.0	25.99	36.69	−0.232	36.06	35.86	0.004
7.0–9.0	44.47	39.60	0.099	40.53	40.20	0.007
<7.0	11.19	9.31	0.062	9.64	9.43	0.007
Missing	18.35	14.40	0.107	13.78	14.52	−0.021
Mean (SD)^b^	8.58 (1.72)	9.00 (1.84)	−0.235	8.97 (1.88)	8.96 (1.83)	0.002
eGFR, ml/min						
≥90	38.16	39.17	−0.021	41.81	40.78	0.021
60–89	32.66	22.77	0.222	24.25	23.82	0.010
30–59	11.59	16.05	−0.130	15.45	15.88	−0.012
<30	0.85	6.71	−0.311	3.47	3.97	−0.026
Missing	16.74	15.30	0.039	15.02	15.54	−0.014
Mean (SD)^b^	84.68 (22.12)	80.64 (30.52)	0.151	84.63 (27.10)	83.00 (28.80)	0.058
LDL−cholesterol, mg/dL						
>140	7.91	8.58	−0.024	8.66	8.53	0.005
120–140	9.32	9.17	0.005	9.47	9.31	0.006
100–119	15.22	15.25	−0.001	15.46	15.28	0.005
<100	44.00	46.91	−0.059	46.79	46.64	0.003
Missing	23.55	20.09	0.084	19.63	20.24	−0.015
Mean (SD)^b^	98.22 (32.15)	97.65 (32.68)	0.018	98.28 (32.56)	97.80 (32.55)	0.015
SBP, mmHg						
>160	3.18	5.24	−0.102	5.05	5.03	0.001
140–160	13.68	19.51	−0.157	19.23	19.35	−0.003
120–139	28.84	41.95	−0.277	42.39	41.81	0.012
<120	9.48	13.48	−0.125	13.91	13.48	0.013
Missing	44.81	19.83	0.554	19.41	20.32	−0.023
Mean (SD)^b^	133.05 (16.47)	133.29 (16.63)	−0.015	133.10 (16.76)	133.18 (16.51)	−0.005
Comorbidities						
Hypertension	66.78	67.05	−0.006	66.00	66.21	−0.004
Ischemic heart disease	24.70	19.33	0.130	18.55	18.92	−0.010
Myocardial infarction	3.33	1.73	0.102	1.55	1.70	−0.012
Coronary artery angioplasty or stenting	2.86	1.49	0.094	1.35	1.53	−0.015
CABG	0.78	0.74	0.005	0.70	0.73	−0.004
Cerebrovascular disease	9.77	8.98	0.027	8.64	8.66	−0.001
Ischemic stroke	6.10	5.27	0.036	5.03	5.12	−0.004
Hemorrhagic stroke	1.39	0.96	0.041	1.07	0.97	0.010
Cardiac dysrhythmia	7.22	5.66	0.064	5.27	5.47	−0.009
Congestive heart failure	8.42	7.59	0.030	7.15	7.12	0.001
Peripheral vascular disease	2.34	4.22	−0.106	4.12	3.92	0.010
Hyperlipidemia	75.49	77.14	−0.039	77.69	77.19	0.012
Chronic kidney disease	8.28	15.39	−0.222	12.63	13.27	−0.019
Charlson comorbidity index, mean (SD)	2.56 (1.75)	2.97 (1.89)	−0.221	2.89 (1.91)	2.89 (1.85)	<0.001
Anti-hyperglycemic medication use						
Any insulin	22.83	56.36	−0.730	54.22	54.13	0.002
Basal insulin	12.27	44.85	−0.774	42.18	42.31	−0.003
Premixed insulin	6.96	16.07	−0.288	14.95	15.15	−0.006
Metformin	89.09	84.77	0.128	87.10	86.57	0.015
Sulfonylurea	55.75	56.89	−0.023	56.76	57.06	−0.006
Glinides	0.93	1.75	−0.071	1.44	1.62	−0.015
Pioglitazone	19.51	20.99	−0.037	21.92	21.02	0.022
α-glucosidase inhibitors	17.29	19.96	−0.069	19.35	19.58	−0.006
DPP-4i	31.92	39.16	−0.152	38.12	37.99	0.003
Number of oral anti-hyperglycemic medications^c^, mean (SD)	2.14 (1.04)	2.24 (1.07)	−0.085	2.25 (1.09)	2.24 (1.07)	0.008
Non- anti-hyperglycemic medication use						
ACEIs or ARBs	61.28	61.88	−0.012	60.39	60.82	−0.009
β blockers	36.39	33.92	0.052	32.36	32.87	−0.011
Calcium channel blockers	27.87	28.60	−0.016	27.48	27.62	−0.003
Diuretics	14.86	19.42	−0.121	17.37	17.85	−0.013
Other anti-hypertensive agents	5.08	6.59	−0.065	5.86	5.83	0.001
Nitrates	13.52	11.37	0.065	10.68	10.94	−0.008
Ivabradine	0.37	0.16	0.040	0.19	0.16	0.009
Valsartan + sacubitril	0.42	0.19	0.041	0.22	0.18	0.008
Aldactone	4.46	4.40	0.003	3.99	4.10	−0.006
Eplerenone	0.11	0.08	0.011	0.10	0.07	0.010
Anti-arrhythmic agents	3.36	2.88	0.027	2.75	2.87	−0.007
Digoxin	1.66	1.32	0.028	1.47	1.32	0.013
Aspirin	32.42	29.65	0.060	28.33	28.97	−0.014
Clopidogrel	8.32	6.34	0.076	5.81	6.16	−0.015
Warfarin	1.04	1.02	0.002	1.02	0.97	0.005
New oral anticoagulant	2.27	1.33	0.071	1.28	1.32	−0.003
Statins	62.62	61.59	0.021	61.09	61.29	−0.004
Fibrates	13.15	14.45	−0.037	14.64	14.33	0.009
Number of cardiovascular-related medications^d^, mean (SD)	2.84 (1.91)	2.80 (1.96)	0.018	2.71 (1.93)	2.73 (1.93)	−0.014
Healthcare utilization						
Echocardiography	11.99	10.41	0.050	9.49	9.96	−0.016
Carotid ultrasonography	3.67	3.46	0.011	3.42	3.39	0.001
Transcranial ultrasonography %	2.37	2.10	0.018	2.12	2.09	0.002
Lower extremity arterial ultrasonography	0.94	1.44	−0.046	1.35	1.35	−0.001
24-h ECG examination	2.45	2.18	0.018	1.98	2.06	−0.005
BNP, proBNP, or NT-proBNP test	4.85	5.26	−0.019	4.79	4.93	−0.006
Prescriber’s specialty						
Cardiologist or cardiovascular surgeon	23.82	4.75	0.566	4.40	4.95	−0.026
Endocrinologist	35.06	67.74	−0.692	67.52	66.92	0.013
Other specialty	41.12	27.51	0.290	28.08	28.14	−0.001
Number of hospitalizations, mean (SD)	0.21 (0.53)	0.29 (0.62)	−0.150	0.28 (0.64)	0.28 (0.60)	<0.001
Number of hospitalization due to CV-related episodes, mean (SD)	0.16 (0.47)	0.21 (0.53)	−0.105	0.20 (0.55)	0.20 (0.51)	−0.004
Number of hospitalization due to genito-urinary infection-related episodes, mean (SD)	0.02 (0.17)	0.04 (0.22)	−0.076	0.04 (0.22)	0.04 (0.21)	0.007
Number of hospitalization due to diabetic ketoacidosis, mean (SD)	0.00 (0.05)	0.00 (0.07)	−0.039	0.00 (0.07)	0.00 (0.07)	0.001
Number of outpatient visits, mean (SD)	17.67 (11.10)	19.54 (12.09)	−0.161	19.23 (12.16)	19.27 (11.94)	−0.003
Number of outpatient visits due to CV−related episodes, mean (SD)	7.47 (5.02)	7.98 (5.66)	−0.096	7.83 (5.49)	7.83 (5.55)	−0.001
Number of outpatient visits due to genito-urinary infection-related episodes, mean (SD)	0.37 (1.44)	0.52 (1.81)	−0.091	0.50 (1.72)	0.50 (1.77)	−0.003

C statistics for PS model: 0.814.

a Data presented as percentage unless otherwise specified (SD, standard deviation).

b Statistics among patients without missing value.

c Oral anti-hyperglycemic medications as listed above.

d Cardiovascular-related medications as listed above.

Abbreviations: ACEIs, angiotensin-converting enzyme inhibitors; ARBs, angiotensin II receptor blockers; BNP, B-type natriuretic peptide; CABG, coronary artery bypass graft surgery; CV, cardiovascular; ECG, electrocardiogram; NT, N-terminal (Abbreviations that have been defined in the main text are not listed here).

A nearest-neighbor algorithm without replacement was used to perform 1:1 match between SGLT-2i and GLP-1RA initiators. The process allowed a maximum matching caliper of 0.025 on the PS scale. For each covariate, the standardized difference less than 0.1 was regarded as well balance between treatment groups (Austin, 2011).

Cox proportional hazards models were used to estimate hazard ratios (HRs) and 95% confidence intervals (CIs) for each outcome with SGLT-2i as compared with GLP-1RA in the eligible cohort before and after PS matching. Kaplan-Meier curves were plotted to demonstrate curves of cumulative incidence of the outcome over time among PS-matched cohorts. Schoenfeld residual tests were used to assess the proportional-hazards assumption.

### Auxiliary and subgroup analyses

New users of dipeptidyl peptidase-4 inhibitors (DPP-4i) was chosen as a second active comparator group to address the comparative risk of SGLT-2i use with other anti-diabetes agents on hospitalized CLI and LEA (See Supplementary Figure S1 for criteria and assembly of the second cohort comparing SGLT-2i with DPP-4i). Subgroup analyses were performed according to age (>60 and <60 years), the presence of established CVD, previous diagnosis of chronic kidney disease (CKD) or baseline eGFR<60 ml/min/1.73 m^2^. Effect modification was assessed according to these prespecified risk factors and examined by looking at overlap of the 95% confidence intervals between subgroups.

In auxiliary analyses, HRs for acute limb events were evaluated after excluding patients with peripheral vascular disease or CLI or LEA at baseline to avoid misclassifying underlying disease as outcome.

Finally, the risks of acute limb events associated with the use of empagliflozin and dapagliflozin was estimated separately. We re-estimated the PS and re-matched patients for each pairwise comparison in each auxiliary and subgroup analysis. All analyses were performed with SAS software, version 9.3 (SAS Institute). All reported *p* values are two-sided.

## Results

### Study population

**FIGURE 1:**
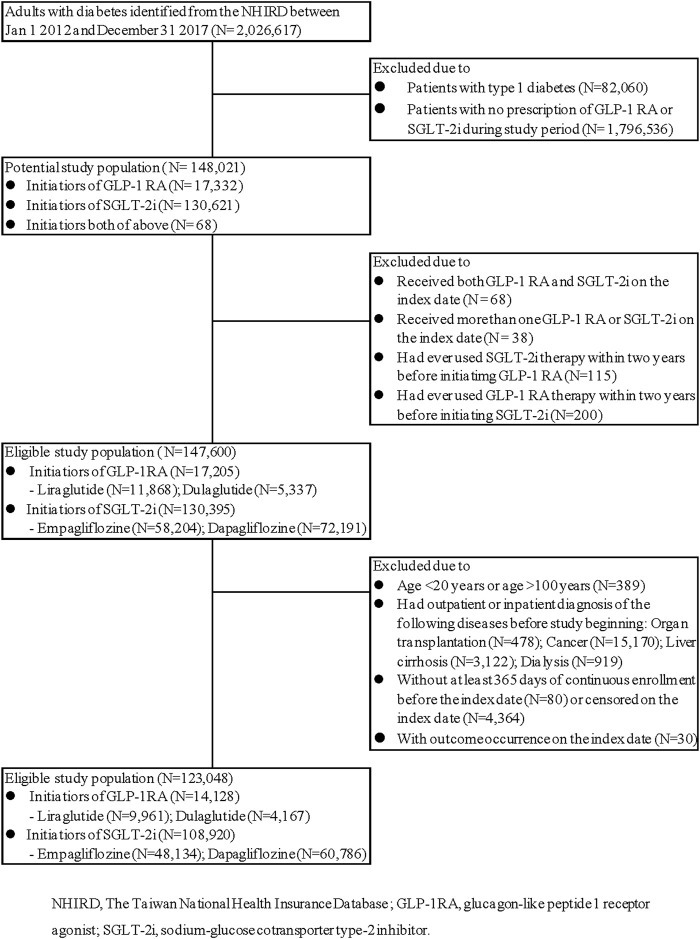
Study cohort assembly. (SGLT-2i versus GLP-1RA).

Out of nearly two million type 2 diabetes adults, a total of 123,048 patients fulfilled the inclusion criteria, including 108,920 SGLT-2i and 14,128 GLP-1RA initiators (Figure 1).

Before PS matching, the patients who initiated SGLT-2i were older, more likely to be men, more likely to have ischemic heart disease or myocardial infarction, and more likely to have received a filled prescription for metformin; GLP-RA initiators had higher HbA1c level, had higher Charlson comorbidity index, were more likely to have diagnosis associated with obesity and CKD, and were more like to receive a filled prescription for insulin, DPP-4i and diuretics (Table 1, left). After PS matching, there were 13,378 SGLT-2i and 13,378 GLP-1RA initiators. The standard difference in each covariate was lower than 0.1, indicating that both groups were well balanced in various baseline characteristics. (Table 1, right).

### Incidence and risk of critical limb ischemia and lower extremity amputation associated with SGLT-2i versus GLP-1RA.

**FIGURE 2:**
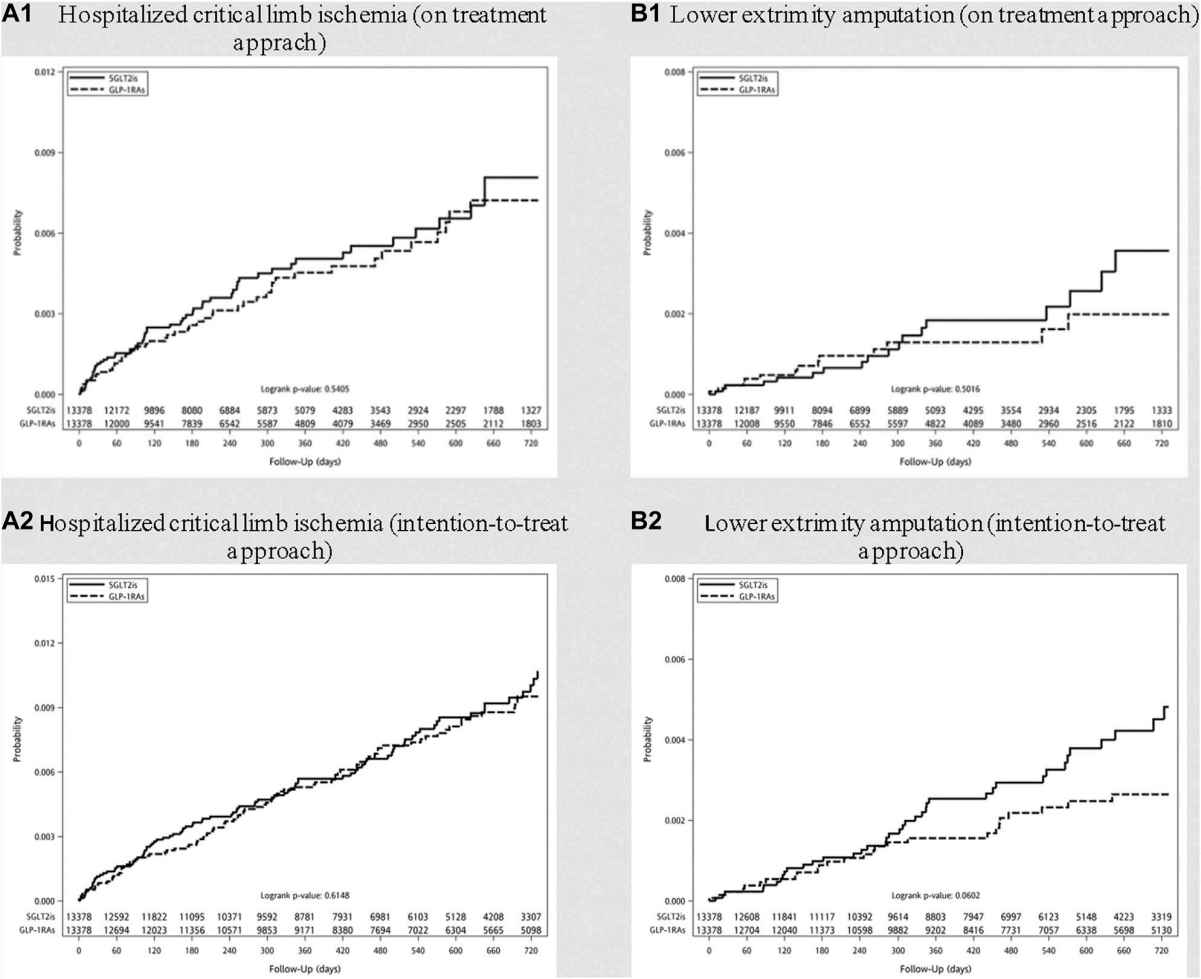
Cumulative incidence curves of **(A)** hospitalized critical limb ischemia and **(B)** lower extremity amputation among diabetes patients initiating sodium-glucose cotransporter type-2 inhibitors (SGLT-2i) and glucagon-like peptide 1 receptor agonists (GLP-1RA) after propensity score matching. (On treatment approach: A1 and B1; Intention-to-treat approach: A2 and B2).

The median follow-up time in SGLT-2i and GLP-1RA groups were 0.62 and 0.67 years, corresponding to crude incidence of 4.00 (95% CI, 3.62–4.43) and 4.52 (95% CI, 3.46–5.90) per 1,000 person-years for hospitalization for CLI; and 1.12 (95% CI, 0.92–1.35) and 1.25 (95% CI, 0.76–2.08) per 1,000 person-years for LEA respectively. The incidence rates for major adverse limb events using either on-treatment or intention-to-treat approach, before and after PS matching, were listed in Supplementary Table S7. The cumulative incidence curves for hospitalized CLI and LEA after PS matching were shown in Figure 2.

Hazards ratios of hospitalization for CLI and LEA among new users of SGLT-2i, as compared with GLP-1RA, are shown in Table 2. After PS matching, the use of SGLT-2i was not associated with an increased risk for hospitalized CLI and LEA in comparison with GLP-1RA (HR 1.13; 95% CI, 0.77–1.65 and 1.27; 95% CI, 0.63–2.55 respectively). A lack of significantly increased risks for CLI and LEA was also noted in the analyses of intention-to-treat approach among SGLT-2i as compared with GLP-1RA initiators.

**TABLE 2:** Hazard ratios of hospitalization for critical limb ischemia and lower extrimity amputation comparing sodium-glucose cotransporter type-2 inhibitor (SGLT-2i) versus glucagon-like peptide 1 receptor agonist (GLP-1RA) initiators.

	GLP-1RA	SGLT-2i	
		Hazard ratio (95% CI)	
		On-treatment approach	Intention-to-treat approach
Hospitalization for critical limb ischemia			
Crude	Reference	0.88 (0.66–1.17)	0.78 (0.63–0.96)
After PS matching	Reference	1.13 (0.77–1.65)	1.08 (0.81–1.44)
Lower extremity amputation			
Crude	Reference	0.89 (0.51–1.52)	0.86 (0.58–1.28)
After PS matching	Reference	1.27 (0.63–2.55)	1.60 (0.98–2.63)

GLP-1RA, glucagon-like peptide 1 receptor agonist; SGLT-2i, sodium-glucose cotransporter type-2 inhibitor; CI, confidence interval; PS, propensity score.

### Findings in subgroups and auxiliary analyses

Table 3 presents the risks of adverse limb events in subgroups of patients stratified according to age, CVD, and CKD. None of these prespecified patients’ baseline characteristics were considered effect modifiers. Consistent with the main analysis, patients taking SGLT-2i, in comparison with GLP-1RA users, did not have higher risks for hospitalized CLI and LEA between patients aged less than or higher than 60 years, and between patients with or without CKD. However, a trend of higher risks for LEA were noted among SGLT-2i users with history of CVD (HR, 2.16; 95% CI, 0.82–5.68). In auxiliary analysis, risk estimates did not change substantially when excluding patients with prior history of peripheral vascular disease (Supplementary Table S8). Risks of CLI and LEA were similar between empagliflozin and dapagliflozin when comparing with GLP-1RA (Supplementary Table S9; Supplementary Figure S2).

**TABLE 3:** Subgroup analyses: hazard ratios of hospitalization for critical limb ischemia and lower extrimity amputation stratified by age, cardiovascular disease and chronic kidney disease.

	N =	GLP-1RA	SGLT-2i
			Hazard ratio (95% CI)
Hospitalization for critical limb ischemia			
Age (years)			
≤60	17,932	Reference	1.11 (0.59–2.10)
>60	8,780	Reference	1.10 (0.68–1.79)
Cardiovascular diseases			
Yes	8,636	Reference	1.56 (0.97–2.51)
No	18,118	Reference	1.19 (0.64–2.22)
Chronic kidney disease			
Yes or eGFR<60 ml/min/1.73m^2^	6,654	Reference	1.42 (0.74–2.70)
No or eGFR ≥ 60	20,032	Reference	1.10 (0.67–1.82)
Lower extremity amputation			
Age (years)			
≤60	17,932	Reference	1.30 (0.49–3.50)
>60	8,780	Reference	0.83 (0.28–2.47)
Cardiovascular diseases			
Yes	8,636	Reference	2.16 (0.82–5.68)
No	18,118	Reference	1.54 (0.60–3.97)
Chronic kidney disease			
Yes or eGFR<60 ml/min/1.73m^2^	6,654	Reference	1.04 (0.26–4.16)
No or eGFR ≥ 60	20,032	Reference	1.16 (0.50–2.68)

GLP-1RA, glucagon-like peptide 1 receptor agonist; SGLT-2i, sodium-glucose cotransporter type-2 inhibitor; CI, confidence interval; eGFR, estimated glomerular filtration.

### Risk of critical limb ischemia and lower extremity amputation associated with SGLT-2i versus DPP-4i

A separate cohort was then recruited to evaluate the risks of SGLT-2i-assciated adverse limb events using DPP-4i as a second active comparator (Supplementary Figure S1). Patients initiating DPP-4i were older and had higher Charlson co-morbidity index scores, more inpatient and outpatient health service utilization, and higher percentage of CKD or eGFR<30 ml/min as compared with SGLT-2i (Supplementary Table S10). DPP-4i users also had meaningfully higher cardiovascular burden in terms of more cardiovascular-related diseases, medications use, and higher cardiovascular-related healthcare access. During follow-up, substantially lower crude incidence rates for hospitalized CLI and LEA were noticed in SGLT-2i users in comparison with DPP-4i (Supplementary Table S11). However, after PS matching, the effect estimates of SGLT-2i versus DPP-4i for adverse limb events shifted dramatically from 0.41 to 1.06, suggesting a high probability of confounding. (Supplementary Table S12; Supplementary Figure S3). As compared with DPP-4i use, SGLT-2i use was not associated with significantly altered risks for hospitalized CLI and LEA, and consistent results were shown in subgroup analysis (Supplementary Table S13). However, potential higher risks for LEA were also noted in SGLT-2i, as compared with DPP-4i, among patients with CVD (HR, 2.06; 95%, CI 0.81–5.25).

## Discussion

The results from this nationwide cohort study indicated that initiating SGLT-2i was not associated with significantly altered risks for hospitalized CLI and LEA as compared with initiating GLP-1RA. There was no association with lower limb adverse events either when the comparison was made between SGLT-2i and DPP-4i initiators. Although the study might be underpowered to explore potential effect modification, a trend of higher risks for LEA was noted among SGLT-2i users with CVD as compared with either GLP-1RA or DPP-4i.

One of the potential mechanisms of action for adverse limb events associated with SGLT-2i use is its diuretic effect that leads to hemoconcentration and increased blood viscosity, making poorly perfused peripheral tissue more prone to ischemia. However, some evidence derived from clinical trials did not suggest an increased risk when SGLT-2i was compared with placebo or other antidiabetic drugs (Inzucchi et al., 2018; Li et al., 2018; Scheen, 2018; Perkovic et al., 2019; Miyashita et al., 2020). With regard to real-world data comparing GLT-1RA and SGLT-2i, results were conflicting. Using registries from Denmark and Sweden, Ueda et al. (2018) reported a 2.32-fold increase in risk for LEA among SGLT-2i users compared with GLP-1RA ones. The work by Fralick et al. (2020) classified participants from three large U.S. health insurance databases into four groups according to whether they were older or younger than 65 years and whether they had or did not have CVD. It turned out that patients prescribed with canagliflozin had a significantly higher risk of LEA than those for whom GLP-1RA were prescribed (HR: 1.73, 95% CI: 1.30–2.29) only in the subgroup older than 65 years with coexisting CVD (Fralick et al., 2020). However, other studies from the United States showed that SGLT-2i, as compared to GLP-1RA, posed a non-statistically significant risk to major adverse limb events (Chang et al., 2018) or was associated with an increased risk only among older adults (Patorno et al., 2021). Paul et al. (2021) reported that the risk of LEA was not higher in SGLT-2i (including 46% canagliflozin users) versus GLP1-RA but lower while compared with DPP-4i. A recent multi-institutional study in Taiwan reported a 38% reduction in major adverse limb events among initiators of GLP-1RA as compared with SGLT-2i, majorly empagliflozin and dapagliflozin, suggesting a detrimental effect of SGLT-2i (Hsiao et al., 2021). Our study found that treatment with SGLT-2i, empagliflozin or dapagliflozin, as compared with treatment with GLP-1RA, was not associated with increased risk for CLI and LEA. However, as seen in subgroup analysis, a potential increased risk for LEA could be speculated among patients with baseline CVD. Since only a small percentage of population had established PAD or CVD at baseline, further studies focusing on high-risk patients are needed. Whether the risk of lower limb adverse events associated with SLGT-2i use is a class effect or drug specific remains uncertain.

Previous studies had suggested strong similarity among SGLT-2i and GLP-1RA initiators in terms of age and cardiovascular risk profiles, which are two crucial risk factors for lower limbs adverse events (Fralick et al., 2019; Fralick et al., 2020), and a similar pattern was found in our study as well. Meanwhile, initiators of DPP-4i in our study, the second active comparator, were older, more likely to have poor renal function, and associated with more cardiovascular related burden at baseline than those of SGLT-2i, in agreement with that reported in previous studies (Fralick et al., 2019; Patorno et al., 2019). Effect measures of SGLT-2i for lower limb adverse events changed considerably from protective to close to null after PS matching, indicating great confounding effect by baseline characteristics among comparison groups. Although we found a marginal negative association between SGLT-2i use and lower limb adverse events while comparing with DPP-4i use, biases from residual confounding remain a notable issue for consideration.

Strengths of this study are as followed. The outpatient pharmacy claims database contains almost all anti-diabetics prescriptions dispensed in Taiwan with high validity for drugs exposure. The complete follow-up of the NHIRD beneficiaries avoids potential selection bias encountered in hospital-based studies. We included a comprehensive list of inpatient diagnostic and procedure codes as well as health insurance reimbursement codes for lower limb revascularization and amputation in order to have more complete outcome ascertainment. In addition, most prior related studies were only claims-based. The current study incorporated crucial clinical variables of which the percentage of missing data was very low into the process of PS matching. The baseline characteristics of the two comparison groups could be closely matched so that the distribution of risk factors and the severity of diabetes and CVD were highly comparable. Since PS matching of variables or proxies associated with disease severity could achieve balance for unmeasured characteristics and minimize unmeasured confounding factors (Patorno et al., 2018), potential confounding by indication was largely mitigated.

Our study has several limitations. First, despite having controlled for a large number of potential confounders and clinical parameters, we could not exclude the possibility of residual confounding, such as body mass index (BMI) or duration of smoking. Second, because the continuous treatment rates of the study drugs were low and it was not until May 2016 that SGLT-2i was reimbursed by NHI, the length of follow-up was limited; the median time of follow-up was 0.62–0.67 year for the on-treatment approach and extended to 1.23–1.53 year for intention-to-treat approach. However, since the diuretic effect of SGLT-2i, which is postulated as the mechanism for lower limb adverse events, is more evident in the early phase of treatment, we were able to capture the short-term risks, if any, of the drug (Vlachopoulos et al., 2021). On the other hand, whether the use of SGLT-2i in the long run would modify the course of the development or progression of PAD needs further investigation (Paul et al., 2021). Third, due to a low incidence of the study outcome and the limited number of patients, the results of subgroup analyses might not be precise. Lastly, our study findings were limited to four specific medications that were widely used in Taiwan. We did not further investigate other GLP-1RA or SGLT-2i for their risks on hospitalized CLI or LEA.

In conclusion, the use of SGLT-2i was not associated with the risks for CLI and LEA as compared to that of GLP-1RA. Further studies using larger sample size population, with broader spectrum of cardiovascular profiles and longer period of follow-up are needed to shed light on real-world evidence as well as to guide clinical practice.

## Data Availability

The datasets presented in this article are not readily available due to the data protection policy declared by National Health Insurance Administration, Taiwan. Further inquiries can be directed to the corresponding authors.
