# Supplementary material for: Risk of major adverse limb events in patients with type 2 diabetes mellitus receiving sodium glucose cotransporter 2 inhibitors and glucagon-like peptide-1 receptor agonists: A population-based retrospective cohort study
Source: Front Pharmacol. 2022 Sep 13;13:869804. doi: 10.3389/fphar.2022.869804 (PMC9513310; doi:10.3389/fphar.2022.869804)
Supplement: Supplementary file 1 [file DataSheet1.docx]

**Risk of major adverse limb events in patients with type 2 diabetes mellitus receiving sodium glucose cotransporter 2 inhibitors and glucagon-like peptide-1 receptor agonists: a population-based retrospective cohort study**

**Supplementary materials**

**List of Tables**

[Supplementary Table 1 International Classification of Diseases, 9th or 10th Revision, Clinical Modification (ICD-9-CM or ICD-10-CM) diagnosis codes used to identify diabetic patients and Anatomical Therapeutic Chemical (ATC) classification system codes used to identify use of study drugs. 4](#_Toc102858323)

[Supplementary Table 2 International Classification of Diseases, 9th or 10th Revision, Clinical Modification (ICD-9-CM or ICD-10-CM) diagnosis or procedure codes or aiwan National Health Insurance reimbursement codes used to exclude patients with vulnerable medical diseases 5](#_Toc102858324)

[Supplementary Table 3 International Classification of Diseases, 9^th^ or 10^th^ Revision, Clinical Modification (ICD-9-CM or ICD-10-CM) diagnosis or procedure codes^a^ and Taiwan National Health Insurance reimbursement codes used to identify the outcomes of interest 7](#_Toc102858325)

[Supplementary Table 4 International Classification of Diseases, 9^th^ or 10^th^ Revision, Clinical Modification (ICD-9-CM or ICD-10-CM) diagnosis or procedure codes^a^ and Taiwan National Health Insurance reimbursement codes^b^ used to identify smoking status, comorbidities, and healthcare utilization at baseline 10](#_Toc102858326)

[Supplementary Table 5 Anatomical Therapeutic Chemical (ATC) classification system codes and Taiwan National Health Insurance reimbursement codes used to identify medications use^a^ at baseline 15](#_Toc102858327)

[Supplementary Table 6 Measurement of the four clinical parameters at baseline 17](#_Toc102858328)

[Supplementary Table 7 Follow-up duration, number of cases, and incidence of critical limb ischemia and lower extremity amputation among propensity score-matched cohort of sodium-glucose cotransporter type-2 inhibitor (SGLT-2i) and glucagon-like peptide 1 receptor agonist (GLP-1 RA) initiators before and after propensity score (PS) matching. 18](#_Toc102858329)

[Supplementary Table 8 Auxiliary analyses: Hazard ratios of hospitalization for critical limb ischemia and lower extremity amputation comparing SGLT-2i initiators versus GLP-1 RA initiators after excluding patients with peripheral vascular disease or critical limb ischemia or lower extremity amputation at baseline - After propensity score matching 20](#_Toc102858330)

[Supplementary Table 9 Auxiliary analyses: Hazard ratios of hospitalization for critical limb ischemia and lower extremity amputation comparing empagliflozin and dapagliflozin initiators versus glucagon-like peptide 1 receptor agonist (GLP-1 RA) initiators - After propensity score matching. (On-treatment approach) 21](#_Toc102858331)

[Supplementary Table 10 Demographics, smoking, clinical parameters, comorbidities, other medication use, and healthcare utilization at baseline among initiators of sodium glucose cotransporter 2 inhibitors (SGLT2i) and dipeptidyl peptidase-4 inhibitor (DPP-4i) before and after PS matching 22](#_Toc102858332)

[Supplementary Table 11 Follow-up duration, number of cases, and incidence of critical limb ischemia and lower extrimity amputation among sodium-glucose cotransporter type-2 inhibitor (SGLT-2i) and dipeptidyl peptidase-4 inhibitor (DPP-4i) initiators (a) before and (b) after propensity score matching. 28](#_Toc102858333)

[Supplementary Table 12 Hazard ratios of hospitalization for critical limb ischemia and lower extrimity amputation comparing sodium-glucose cotransporter type-2 inhibitor (SGLT-2i) initiators versus dipeptidyl peptidase-4 inhibitor (DPP-4i) initiators. (N = 589,327) 30](#_Toc102858334)

[Supplementary Table 13 Subgroup analyses: hazard ratios of hospitalization for critical limb ischemia and lower extrimity amputation comparing sodium-glucose cotransporter type-2 inhibitor (SGLT-2i) initiators versus dipeptidyl peptidase-4 inhibitor (DPP-4i) initiators stratified by age, with or without cardiovascular disease, and chronic kidey disease. 31](#_Toc102858335)

**List of Figures**

[Supplementary Figure 1 Study cohort assembly (SGLT-2i versus DPP-4i). 32](#_Toc94948738)

[Supplementary Figure 2 Cumulative incidence curves of hospitalized critical limb ischemia and lower extremity amputation among diabetes patients initiating empagliflozin and those initiating dapagliflozin versus glucagon-like peptide 1 receptor agonist (GLP-1RA) use after propensity score matching. 34](#_Toc94948739)

[Supplementary Figure 3 Cumulative incidence curves of (a) hospitalized critical limb ischemia and (b) lower extremity amputation among diabetes patients initiating sodium-glucose cotransporter type-2 inhibitors (SGLT-2i) and dipeptidyl peptidase-4 inhibitors (DPP-4i) after propensity score matching. (On treatment approach: a1 and b1; Intention-to-treat approach: a2 and b2) 36](#_Toc94948740)

Supplementary Table 1 International Classification of Diseases, 9th or 10th Revision, Clinical Modification (ICD-9-CM or ICD-10-CM) diagnosis codes used to identify diabetic patients and Anatomical Therapeutic Chemical (ATC) classification system codes used to identify use of study drugs.

| **Diagnosis** | **Diagnosis codes** |
| --- | --- |
| Diabetes | ICD-9-CM diagnosis codes: 250  ICD-10-CM diagnosis codes: E08, E09, E10, E11, or E13 |
| **Study drugs** | **ATC classification system codes** |
| **GLP-1RA** | |
| Liraglutide | A10BJ02  Adult dosage: 0.6-1.8 mg once weekly  Dosage form and strength: 18 mg/3 mL/penfill (multiple injectable doses)  First reimbursement date in Taiwan: October 1, 2012 |
| Dulaglutide | A10BJ05  Adult dosage: 0.75 or 1.5 mg once weekly (may increase to a maximum of 4.5 mg once weekly)  Dosage form and strength: 0.75 or 1.5 mg/0.5 mL/penfill (single injectable dose)  First reimbursement date in Taiwan: May 1, 2016 |
| **SGLT2i** | |
| Dapagliflozin | A10BK01  Adult dosage: 5 or 10 mg once daily  Dosage form and strength: 5 or 10 mg/tablet  First reimbursement date in Taiwan: May 1, 2016 |
| Empaglifozin | A10BK03  Adult dose: 10 or 25 mg once daily  Dosage form and strength: 10 or 25 mg/tablet  First reimbursement date in Taiwan: May 1, 2016 |

GLP-1RA, glucagon-like peptide-1 receptor agonists; SGLT2i, sodium glucose cotransporter 2 inhibitor.

^a^Based on inpatient and outpatient pharmacy dispensing claims.

Supplementary Table 2 International Classification of Diseases, 9th or 10th Revision, Clinical Modification (ICD-9-CM or ICD-10-CM) diagnosis or procedure codes or aiwan National Health Insurance reimbursement codes used to exclude patients with vulnerable medical diseases

| **Underlying diseases** | **Diagnosis or procedure codes or Taiwan health insurance service claims codes** |
| --- | --- |
| Organ transplantation | ICD-9-CM diagnosis codes: 996.8, V042  ICD-10-CM diagnosis codes: D89.81, T86, Z94 |
| Cancer | ICD-9-CM diagnosis codes: 140-208, 230-234, 511.81, 789.51  ICD-10-CM diagnosis codes: C00-C26, C30-C41, C43-C58, C60-C72, C73-C75**,** D00-D09, J91.0, R18.0, Z51.0, Z51.1 |
| Liver cirrhosis | ICD-9-CM diagnosis codes: 571.2, 571.5, 571.6  ICD-10-CM diagnosis codes: K70.2, K70.3, K74 |
| Dialysis | ICD-9-CM diagnosis codes: V45.1, V56.0, V56.8  ICD-10-CM diagnosis codes: Z49.3, Z99.2  ICD-9-CM procedure codes: 39.95, 54.98  ICD-10-CM procedure codes: 5A1D00Z, 5A1D60Z, 3E1M39Z  Taiwan health insurance service claims codes: 58001C, 58002C, 58009B, 58010B, 58011C, 58012B, 58013C, 58017C, 58018C, 58026C, 58027C, 58028C, 58029C, 58030B, 69006C, ACPD1MTC01M0, CGDW1960012C, CGDW1MSU70MW, CGS01440042C, CGS01440132C, CKD006752L2X, CKD006753L2X, CKDD1135132C, CKDD1170052C, CKDD117005KD, CKDD1170092C, CKDD117009KD, CKDD122334BQ, CKDD124334BQ, CKDD1390032C, CKDD139003KD, CKDD154334BQ, CKDD1881CK2C, CKDD1881SK2C, CKDD1883CK2C, CKDD1883SK2C, CKDD1C0122AR, CKDD1C2122AR, CKDD1M2122AR, CKDD1XTP11M0, CKDD1XTP94M0, CKDD210598SB, CKDD2140012C, CKDD214001KD, CKDD2140022C, CKDD214002KD, CKDD214554NL, CKDD2330742C, CKDD2420052C, CKDD242005KD, CKDD2450072C, CKDD245007KD, CKDD245540SB, CKDD245542SB, CKDD2480012C, CKDD257331BA, CKDD2690012C, CKDD269001KD, CKDD269033BA, CKDD2881452C, CKDD288155KD, CKDD2SL12PM0, CKDD2SL18PM0, CKDD323334BQ, CKDD3456032C, CKDD345603KD, CKDD3C2123AR, CKDD3M2123AR, CKDD3XTP31M0, CKP010T2022C, CKP018812N2C, CKP018812NKD, CKP018814N2C, CKP018814NKD, CKP018817N2C, CKP018817NKD, CKP018880N2C, CKP018880NKD, CKP018884N2C, CKP018888N2C, CKP018890N2C, CKP018890NKD, CKP01MC20XM0, CKP01MC232M0, CKP022C410BT, CKP0413836FA, CKP04C4407BT, CKP04C4479BT, CKP04C8303BT, CPC0260048DV, FUK0500M60GA, FUK050M100GA, FUK05EMKC2FA, FUK05ST100GA, FUK05ST60PGA, FUK05TS404FN, FUK05TS413FN, HEF03PRFPEGA, HEF03PRLPEGA, HEF03PRTPEGA |

Supplementary Table 3 International Classification of Diseases, 9^th^ or 10^th^ Revision, Clinical Modification (ICD-9-CM or ICD-10-CM) diagnosis or procedure codes^a^ and Taiwan National Health Insurance reimbursement codes used to identify the outcomes of interest

| **Outcomes** | **Diagnosis or Procedure codes** |
| --- | --- |
| **Critical limb ischemia**^b^ | **ICD-9-CM diagnosis codes: 440.22, 440.23, 440.24, 443.9x**  **ICD-10-CM diagnosis codes: I70.22, I70.25, I70.26, I73.9** |
|  | **OR any of the following procedure codes for critical limb ischemia** |
|  | **Amputation**  **[Excluding codes for injury or poisoning: ICD-9-CM 800-999 or ICD-10-CM S00-S99, T07-T88))**  **ICD-9-CM procedure codes:**  84.10-84.17 Amputation of lower limb  **ICD-10 procedure codes**  0Y6C0Z1, 0Y6C0Z2, 0Y6C0Z3, 0Y6D0Z1, 0Y6D0Z2, 0Y6D0Z3,  0Y6H0Z1, 0Y6H0Z2, 0Y6H0Z3, 0Y6J0Z1, 0Y6J0Z2, 0Y6J0Z3  **Taiwan National Health Insurance reimbursement codes**  64022B, 64023B, 64024B, 64025C  **Revaasculization**  **ICD-9-CM procedure codes:**  Stenting: 00.55  Bypass: 38.08, 38.18, 38.38, 38.48, 38.68, 38.88, 39.50, 39.7, 39.90, 39.25, 39.26, 39.29  **ICD-10 procedure codes**  Stenting: 047K34Z, 047K3DZ, 047K44Z, 047K4DZ, 047L34Z, 047L3DZ, 047L44Z, 047L4DZ, 047M34Z, 047M3DZ, 047M44Z, 047M4DZ, 047N34Z, 047N3DZ, 047N44Z, 047N4DZ, 047P34Z, 047P3DZ, 047P44Z, 047P4DZ, 047Q34Z, 047Q3DZ, 047Q44Z, 047Q4DZ, 047R34Z, 047R3DZ, 047R44Z, 047R4DZ, 047S34Z, 047S3DZ, 047S44Z, 047S4DZ, 047T34Z, 047T3DZ, 047T44Z, 047T4DZ, 047U34Z, 047U3DZ, 047U44Z, 047U4DZ  Bypass: 041K09H, 041K09J, 041K09K, 041K09L, 041K09M, 041K09N, 041K09P, 041K09Q, 041K09S, 041K0AH, 041K0AJ, 041K0AK, 041K0AL, 041K0AM, 041K0AN, 041K0AP, 041K0AQ, 041K0AS, 041K0JH, 041K0JJ, 041K0JK, 041K0JL, 041K0JM, 041K0JN, 041K0JP, 041K0JQ, 041K0JS, 041K0KH, 041K0KJ, 041K0KK, 041K0KL, 041K0KM, 041K0KN, 041K0KP, 041K0KQ, 041K0KS, 041K0ZH, 041K0ZJ, 041K0ZK, 041K0ZL, 041K0ZM, 041K0ZN, 041K0ZP, 041K0ZQ, 041K0ZS, 041K49H, 041K49J, 041K49K, 041K49L, 041K49M, 041K49N, 041K49P, 041K49Q, 041K49S, 041K4AH, 041K4AJ, 041K4AK, 041K4AL, 041K4AM, 041K4AN, 041K4AP, 041K4AQ, 041K4AS, 041K4JH, 041K4JJ, 041K4JK, 041K4JL, 041K4JM, 041K4JN, 041K4JP, 041K4JQ, 041K4JS, 041K4KH, 041K4KJ, 041K4KK, 041K4KL, 041K4KM, 041K4KN, 041K4KP, 041K4KQ, 041K4KS, 041K4ZH, 041K4ZJ, 041K4ZK, 041K4ZL, 041K4ZM, 041K4ZN, 041K4ZP, 041K4ZQ, 041K4ZS, 041L09J, 041L09K, 041L09L, 041L09M, 041L09N, 041L09P, 041L09Q, 041L09S, 041L0AH, 041L0AJ, 041L0AK, 041L0AL, 041L0AM, 041L0AN, 041L0AP, 041L0AQ, 041L0AS, 041L0JH, 041L0JJ, 041L0JK, 041L0JL, 041L0JM, 041L0JN, 041L0JP, 041L0JQ, 041L0JS, 041L0KH, 041L0KJ, 041L0KK, 041L0KL, 041L0KM, 041L0KN, 041L0KP, 041L0KQ, 041L0KS, 041L0ZH, 041L0ZJ, 041L0ZK, 041L0ZL, 041L0ZM, 041L0ZN, 041L0ZP, 041L0ZQ, 041L0ZS, 041L49H, 041L49J, 041L49K, 041L49L, 041L49M, 041L49N, 041L49P, 041L49Q, 041L49S, 041L4AH, 041L4AJ, 041L4AK, 041L4AL, 041L4AM, 041L4AN, 041L4AP, 041L4AQ, 041L4AS, 041L4JH, 041L4JJ, 041L4JK, 041L4JL, 041L4JM, 041L4JN, 041L4JP, 041L4JQ, 041L4JS, 041L4KH, 041L4KJ, 041L4KK, 041L4KL, 041L4KM, 041L4KN, 041L4KP, 041L4KQ, 041L4KS, 041L4ZH, 041L4ZJ, 041L4ZK, 041L4ZL, 041L4ZM, 041L4ZN, 041L4ZP, 041L4ZQ, 041L4ZS, 041M09L, 041M09M, 041M09P, 041M09Q, 041M09S, 041M0AL, 041M0AM, 041M0AP, 041M0AQ, 041M0AS, 041M0JL, 041M0JM, 041M0JP, 041M0JQ, 041M0JS, 041M0KL, 041M0KM, 041M0KP, 041M0KQ, 041M0KS, 041M0ZL, 041M0ZM, 041M0ZP, 041M0ZQ, 041M0ZS, 041M49L, 041M49M, 041M49P, 041M49Q, 041M49S, 041M4AL, 041M4AM, 041M4AP, 041M4AQ, 041M4AS, 041M4JL, 041M4JM, 041M4JP, 041M4JQ, 041M4JS, 041M4KL, 041M4KM, 041M4KP, 041M4KQ, 041M4KS, 041M4ZL, 041M4ZM, 041M4ZP, 041M4ZQ, 041M4ZS, 041N09L, 041N09M, 041N09P, 041N09Q, 041N09S, 041N0AL, 041N0AM, 041N0AP, 041N0AQ, 041N0AS, 041N0JL, 041N0JM, 041N0JP, 041N0JQ, 041N0JS, 041N0KL, 041N0KM, 041N0KP, 041N0KQ, 041N0KS, 041N0ZL, 041N0ZM, 041N0ZP, 041N0ZQ, 041N0ZS, 041N49L, 041N49M, 041N49P, 041N49Q, 041N49S, 041N4AL, 041N4AM, 041N4AP, 041N4AQ, 041N4AS, 041N4JL, **041N4JM**, 041N4JP, 041N4JQ, 041N4JS, 041N4KL, 041N4KM, 041N4KP, 041N4KQ, 041N4KS, 041N4ZL, 041N4ZM, 041N4ZP, 041N4ZQ, 041N4ZS  **Taiwan National Health Insurance reimbursement codes**  69023B, 33074A, 33074B, 33115B |
| **Lower extremity amputation** | **[Excluding codes for injury or poisoning: ICD-9-CM 800-999 or ICD-10-CM S00-S99, T07-T88))**  **ICD-9-CM procedure codes:**  84.10-84.17 Amputation of lower limb  **ICD-10 procedure codes**  0Y6C0Z1, 0Y6C0Z2, 0Y6C0Z3, 0Y6D0Z1, 0Y6D0Z2, 0Y6D0Z3,  0Y6H0Z1, 0Y6H0Z2, 0Y6H0Z3, 0Y6J0Z1, 0Y6J0Z2, 0Y6J0Z3  **Taiwan National Health Insurance reimbursement codes**  64022B, 64023B, 64024B, 64025C |

^a^Based on any positions on inpatient claims.

^b^ modified from: Bekwelem W, Bengtson LG, Oldenburg NC, Winden TJ, Keo HH, Hirsch AT, Duval S. Development of administrative data algorithms to identify patients with critical limb ischemia. Vasc Med. 2014;19:483–490. ^(^Performance Measure: Sensitivity: 0.92).

Supplementary Table 4 International Classification of Diseases, 9^th^ or 10^th^ Revision, Clinical Modification (ICD-9-CM or ICD-10-CM) diagnosis or procedure codes^a^ and Taiwan National Health Insurance reimbursement codes^b^ used to identify smoking status, comorbidities, and healthcare utilization at baseline

| **Variables** | **Diagnosis or procedure codes or** **Taiwan National Health Insurance reimbursement codes** |
| --- | --- |
| Smoking | ICD-9-CM diagnosis codes: 305.1, 649.0, 989.84, V15.82  ICD-10-CM diagnosis codes: F17.200, F17.201, F17.210, F17.211, F17.220, F17.221, F17.290, F17.291, T65.211A, T65.212A, T65.213A, T65.214A, T65.221A, T65.222A, T65.223A, T65.224A, T65.291A, T65.292A, T65.293A, T65.294A, Z87.891 |
| **Comorbidities** | |
| Overweight or obesity | ICD-9-CM diagnosis codes: 278.0, 278.00, 278.01, 278.02  ICD-10-CM diagnosis codes: E66 |
| Hypertension | ICD-9-CM diagnosis codes: 401-405  ICD-10-CM diagnosis codes: I10-I13, I15 |
| Ischemic heart disease | ICD-9-CM diagnosis codes: 411, 413, 414  ICD-10-CM diagnosis codes: I20, I24, I25 |
| Myocardial infarction | ICD-9-CM diagnosis codes: 410  ICD-10-CM diagnosis codes: I21, I22 |
| Coronary artery angioplasty or stenting | ICD-9-CM procedure codes: 00.66, 36.01, 36.02, 36.05, 36.06, 36.07  ICD-10-CM procedure codes: 02703ZZ, 02713ZZ, 02723ZZ, 02733ZZ  Taiwan health insurance service claims codes: 33076B, 33077B, 33078B |
| CABG | ICD-9-CM diagnosis codes: 414.02, 414.03, 414.04, 414.05  ICD-10-CM diagnosis codes: I25.70, I25.71, I25.72, I25.73, I25.79  ICD-9-CM procedure codes: 36.1, 36.2  ICD-10-CM procedure codes: 0210093, 02100A3, 02100J3, 02100K3, 02100Z3, 021009W, 02100AW, 02100JW, 02100KW, 021109W, 02110AW, 02110JW, 02110KW, 021209W, 02120AW, 02120JW, 02120KW, 0210098, 0210099, 021009C, 02100A8, 02100A9, 02100AC, 02100J8, 02100J9, 02100JC, 02100K8, 02100K9, 02100KC, 02100Z8, 02100Z9, 02100ZC, 0211098, 0211099, 021109C, 02110A8, 02110A9, 02110AC, 02110J8, 02110J9, 02110JC, 02110K8, 02110K9, 02110KC, 02110Z8, 02110Z9, 02110ZC, 0212098, 0212099, 021209C, 02120A8, 02120A9, 02120AC, 02120J8, 02120J9, 02120JC, 02120K8, 02120K9, 02120KC, 02120Z8, 02120Z9, 02120ZC, 021009F, 02100AF, 02100JF, 02100KF, 02100ZF, 021109F, 02110AF, 02110JF, 02110KF, 02110ZF, 021209F, 02120AF, 02120JF, 02120KF, 02120ZF, 0210093, 02100A3, 02100J3, 02100K3, 02100Z3, 0211093, 02110A3, 02110J3, 02110K3, 02110Z3, 0212093, 02120A3, 02120J3, 02120K3, 02120Z3, 0211344, 02113D4  Taiwan health insurance service claims codes: 68023B, 68024B, 68025B, 68053B, 68054B, 68055B, 97901K, 97902A, 97903B, 97906K, 97907A, 97908B, 97911K, 97912A, 97913B, 97916K, 97917A, 97918B, N26002, N26003 |
| Cerebrovascular disease | ICD-9-CM diagnosis codes: 430-438  ICD-10-CM diagnosis codes: G45, G46, I60-I69 (Excluded I67.3, I67.83) |
| Ischemic stroke | ICD-9-CM diagnosis codes: 433, 434, 436  ICD-10-CM diagnosis codes: I63, I65, I66, I67.89 |
| Hemorrhagic stroke | ICD-9-CM diagnosis codes: 430, 431, 432  ICD-10-CM diagnosis codes: I60, I61, I62 |
| Cardiac dysrhythmia/ atrial fibrillation | ICD-9-CM diagnosis codes: 427  ICD-10-CM diagnosis codes: I46-I49, R00.1 |
| Congestive heart failure | ICD-9-CM diagnosis codes: 428, 398.91, 402.01, 402.11, 402.91, 404.01, 404.11, 404.91, 404.03, 404.13, 404.93  ICD-10-CM diagnosis codes: I09.81, I11.0, I13.0, I13.2, I50 |
| Peripheral vascular disease | ICD-9-CM diagnosis codes: 250.7, 440.2, 440.4, 443.81, 443.9, 785.4  ICD-10-CM diagnosis codes: I70.2, I70.92, I73.9, I75.0, I79.1, I79.8, I96 |
| Hyperlipidemia | ICD-9-CM diagnosis codes: 272.0, 272.1, 272.2, 272.3, 272.4, 272.9, 272  ICD-10-CM diagnosis codes: E78.0, E78.1, E78.2, E78.3, E78.4, E78.5, E71.30, E75.21, E75.22, E75.24, E75.3, E75.5, E75.6, E77, E78 (Excluded E78.71, E78.72), E88.1, E88.2, E88.89 |
| Chronic lung disease | ICD-9-CM diagnosis codes: 490-496, 500-508  ICD-10-CM diagnosis codes: J40-J47 (Excluded J45.990), J60-J70 (Excluded J70.5) |
| Chronic kidney disease | ICD-9-CM diagnosis codes: 403.00, 403.01, 403.10, 403.11, 403.90, 403.91, 404.00, 404.01, 404.02, 404.03, 404.10, 404.11, 404.12, 404.13, 404.90, 404.91, 404.92, 404.93, 585, V45.1, V56.0, V56.8  ICD-10-CM diagnosis codes: I12, I13, N18, Z49.3, Z99.2  ICD-9-CM procedure codes: 39.95, 54.98  ICD-10-CM procedure codes: 3E1M39Z, 5A1D00Z, 5A1D60Z |
| Chronic liver disease | ICD-9-CM diagnosis codes: 070.2, 070.3, 070.41, 070.44, 070.51, 070.54, V02.61, V02.62, 571.0, 571.1, 571.2, 571.3, 571.4, 571.5, 571.6, 571.8  ICD-10-CM diagnosis codes: B16, B17.0, B17.1, B18.0, B18.1, B18.2, B19.1, B19.2, K70, K73, K74, K75.4, K75.81, Z22.51, Z22.52 |
| Gastritis or peptic ulcer disease | ICD-9-CM diagnosis codes: 531-535, 578.0, 578.1, 578.9  ICD-10-CM diagnosis codes: K25-K29, K31.82, K52.81, K56.60, K92.0, K92.1, K92.2 |
| Thyroid disease | ICD-9-CM diagnosis codes: 242, 244, 245  ICD-10-CM diagnosis codes: E01, E02, E03, E05, E06, E89.0 |
| Rheumatoid arthritis/ osteoarthritis | ICD-9-CM diagnosis codes: 714, 715, 720  ICD-10-CM diagnosis codes: M05, M06, M08, M12.0, M15-M19, M45, M46.0, M46.1, M46.5, M46.8, M46.9, M48.8, M49.8 |
| Osteoporosis | ICD-9-CM diagnosis codes: 733.0  ICD-10-CM diagnosis codes: M81, M80.00XA, M80.00XK, M80.00XP, M80.011A, M80.011K, M80.011P, M80.012A, M80.012K, M80.012P, M80.019A, M80.019K, M80.019P, M80.021A, M80.021K, M80.021P, M80.022A, M80.022K, M80.022P, M80.029A, M80.029K, M80.029P, M80.031A, M80.031K, M80.031P, M80.032A, M80.032K, M80.032P, M80.039A, M80.039K, M80.039P, M80.041A, M80.041K, M80.041P, M80.042A, M80.042K, M80.042P, M80.049A, M80.049K, M80.049P, M80.051A, M80.051K, M80.051P, M80.052A, M80.052K, M80.052P, M80.059A, M80.059K, M80.059P, M80.061A, M80.061K, M80.061P, M80.062A, M80.062K, M80.062P, M80.069A, M80.069K, M80.069P, M80.071A, M80.071K, M80.071P, M80.072A, M80.072K, M80.072P, M80.079A, M80.079K, M80.079P, M80.08XA, M80.08XK, M80.08XP, M80.80XA, M80.80XK, M80.80XP, M80.811A, M80.811K, M80.811P, M80.812A, M80.812K, M80.812P, M80.819A, M80.819K, M80.819P, M80.821A, M80.821K, M80.821P, M80.822A, M80.822K, M80.822P, M80.829A, M80.829K, M80.829P, M80.831A, M80.831K, M80.831P, M80.832A, M80.832K, M80.832P, M80.839A, M80.839K, M80.839P, M80.841A, M80.841K, M80.841P, M80.842A, M80.842K, M80.842P, M80.849A, M80.849K, M80.849P, M80.851A, M80.851K, M80.851P, M80.852A, M80.852K, M80.852P, M80.859A, M80.859K, M80.859P, M80.861A, M80.861K, M80.861P, M80.862A, M80.862K, M80.862P, M80.869A, M80.869K, M80.869P, M80.871A, M80.871K, M80.871P, M80.872A, M80.872K, M80.872P, M80.879A, M80.879K, M80.879P, M80.88XA, M80.88XK, M80.88XP |
| Gout | ICD-9-CM diagnosis codes: 274  ICD-10-CM diagnosis codes: M10, M1A (excluded M1A.1), N20.0 |
| Dementia | ICD-9-CM diagnosis codes: 290.0-290.4 291.2, 294.1, 294.10, 294.11, 331.0-331.2  ICD-10-CM diagnosis codes: F01, F02, F03.90, F03.91, F05, F10.27, F10.97, G30, G31.0, G31.1, G31.83 |
| **Heathcare utilization** | |
| Echochardiography | Taiwan health insurance service claims codes: 18005B, 18005C, 18006B, 18006C, 18044B |
| Carotid ultrasonography | Taiwan health insurance service claims codes: 20013A, 20013B, 20013C |
| Transcranial ultrasonography | Taiwan health insurance service claims codes: 20026B |
| Lower extremity arterial ultrasonography | Taiwan health insurance service claims codes: 19002BD, 18008B, 18008C |
| 24-hour ECG examination, % | Taiwan health insurance service claims codes: 18019B, 18019C |
| BNP, proBNP, or NT-proBNP test | Taiwan health insurance service claims codes: 12193B, 12193C |
| CV-related episodes | See codes of hypertension, ischemic heart disease, myocardial infarction, congestive heart failure, cerebrovascular disease, peripheral vascular disease, and hyperlipidemia defined above |
| Diabetic ketoacidosis | ICD-9-CM diagnosis codes: 249.1, 250.1  ICD-10-CM diagnosis codes: E08.10, E08.11, E09.10, E09.11, E10.10, E10.11, E11.10, E11.11, E13.10, E13.11 |
| Genito-urinary infection-related episodes | ICD-9-CM diagnosis codes: 590, 595.0, 595.9, 597.0, 597.8, 599.0, 601, 604, 614, 615, 616, 608.4, 682.2, 112.1, 112.2, 112.3  ICD-10-CM diagnosis codes: N10, N11.0, N13.6, N15.1, N30, N34, N39.0, N41, N45, N70, N71, N72, N73, N75.1, N76, N77.1, N49.3, L03.314, L03.315, L03.90, B37.2, B37.3, B37.4 |

BNP, B-type natriuretic peptide; CABG, coronary artery bypass graft surgery; CV, cardiovascular; ECG, electrocardiogram; NT, N-terminal.

^a,b^Based on inpatient and outpatient claims.

^b^<https://www.nhi.gov.tw/query/query2.aspx>. Accessed October 1, 2021.

Supplementary Table 5 Anatomical Therapeutic Chemical (ATC) classification system codes and Taiwan National Health Insurance reimbursement codes used to identify medications use^a^ at baseline

| **Medications** | **ATC classification system codes or Taiwan National Health Insurance reimbursement codes** |
| --- | --- |
| **Anti-diabetic medications** | |
| Insulin | A10A |
| Basal insulin^b^ | Taiwan National Health Insurance reimbursement codes : A034984209, A034984299, B015735209, B015735299, B020310261, B020310266, B020310299, J000110209, J000110299, K000656266, K000657209, K000657299, KC00657209, B017189209, B017189299, K000659209, K000659299, B018836209, B018836299, B019657209, B019657299, K000742209, K000742299, B018994209, B018994299, B020325209, B020325299, B021155209, B021155299, K000697266, K000760209, K000760299, KC00697266, KC00760209, B018823209, B018823299, B018824209, B018824261, B018824299, B018837209, B018837299, B019687209, B019687261, B019687266, B019687299, K000738266, K000738299, B015613209, B015613299, B015806209, B015806299, B019172209, B019172299, K000810266, KC00810266, K000728266, K000745209, KC00728266, KC00745209, KC00986266, KC01011272 |
| Premixed insulin | Taiwan National Health Insurance reimbursement codes: K000595266, K000596266, K000795266, K000898266, K000899266, K000900266, KC00595266, KC00596266, KC00795266, KC00898266, KC00899266, KC00900266, K000768266, K000820266, K000908266, K000909266, KC00768266, KC00820266, KC00908266, KC00909266 |
| Metformin | A10BA02, A10BD02, A10BD03, A10BD05, A10BD07, A10BD08, A10BD10, A10BD11, A10BD13, A10BD15, A10BD16, A10BD20 |
| Sulfonylurea | A10BB, A10BD01, A10BD02, A10BD04, A10BD06 |
| Glinides | A10BX02, A10BX03, A10BX08, |
| Pioglitazone | A10BG03, A10BD05, A10BD06, A10BD09, A10BD12 |
| α-glucosidase inhibitors | A10BF |
| Dipeptidyl peptidase-4 inhibitors | A10BH |
| **Non- anti-diabetic medications** | |
| ACEIs or ARBs | C09 |
| Β blockers | C07 |
| Calcium channel blockers | C08 |
| Diuretics | C03, C07B, C07C, C07D, C08G |
| Other anti-hypertensive agents | C02 |
| Nitrates | C01DA |
| Ivabradine | C01EB17 |
| Valsartan+sacubitril | C09DX04 |
| Aldactone | C03DA01 |
| Eplerenone | C03DA04 |
| Anti-arrhythmic agents | C01B |
| Digoxin | C01AA |
| Aspirin | B01AC06, N02BA01 |
| Clopidogrel | B01AC04 |
| Warfarin | B01AA03 |
| New oral anticoagulants | B01AE, B01AF |
| Statins | C10AA |
| Fibrates | C10AB |
| Urate-lowering agents | M04A |
| Febuxostat | M04AA03 |
| Thyroid-therapy drugs | H03 |
| Systematic corticosteroids | H02AA02, H02AB02, H02AB04, H02AB06, H02AB10, H02BX, H02BX91 |
| Antibiotics | J01 |
| Histamine 2 antagonist or PPI | A02BA, A02BC |
| Anti-epileptics | N03 |
| Anti-depressants | N06A |
| Anti-psychotics | N05A |
| Anxiolytics | N05B |
| Hypnotics | N05C |

ACEIs, angiotensin-converting enzyme inhibitors; ARBs, angiotensin Ⅱ receptor blockers; PPI, proton pump inhibitors.

^a^Based on inpatient and outpatient pharmacy dispensing claims.

^b^Basal insulin included intermediate/long-acting insulin (insulin protamine and insulin isophane) and long-acting insulin (insulin detemir and insulin glargine).

Supplementary Table 6 Measurement of the four clinical parameters at baseline

| **Clinical parameters** | **Assessment period before the cohort entry date^a^** | **Acceptable range^b^** |
| --- | --- | --- |
| HbA1c | 0 to 90 days | No restriction |
| eGFR | 0 to 180 days | 0.2-1.2 mg/dL for serum creatinine (see note below) |
| LDL-cholesterol | 0 to 180 days | 40-250 mg/dL |
| SBP | 0 to 180 days | 70-220 mmHg |
| Note: eGFR was estimated based on information on age, sex, and serum creatinine measured within 0 to 180 days before cohort entry derived from the CKD-EPI formula:^1^  eGFR = 141 × min (S_cr_ /κ, 1)^α^ × max(S_cr_ /κ, 1)^-1.209^ × 0.993^Age^ × 1.018 [if female] × 1.159 [if black]  κ is 0.7 for females and 0.9 for males,  α is -0.329 for females and -0.411 for males,  min indicates the minimum of serum creatinine /κ or 1, and         max indicates the maximum of serum creatinine /κ or 1. | | |

eGFR, estimated glomerular filtration rate; HbA1c, glycated hemoglobin; LDL, low-density lipoprotein; SBP, systolic blood pressure.

^a^If there were more than two values available within the assessment period, the last record was used for the analysis.

^b^The records of each parameter were excluded if the values were beyond the acceptable range.

^1^ (Reference) Levey AS, Stevens LA, Schmid CH, Zhang YL, Castro 3^rd^ AF, Feldman HI; CKD-EPI (Chronic Kidney Disease Epidemiology Collaboration). A new equation to estimate glomerular filtration rate. Ann Intern Med. 2009;150:604-61

Supplementary Table 7 Follow-up duration, number of cases, and incidence of critical limb ischemia and lower extremity amputation among propensity score-matched cohort of sodium-glucose cotransporter type-2 inhibitor (SGLT-2i) and glucagon-like peptide 1 receptor agonist (GLP-1 RA) initiators before and after propensity score (PS) matching.

1. **Before propensity score matching**

|  | SGLT-2i initiators  ( N= 108,920) | GLP-1 RA initiators  ( N=14,128) |
| --- | --- | --- |
|  |  |  |
| **On-treatment approach** |  |  |
| **Hospitalization for critical limb ischemia** |  |  |
| Total follow-up person-years | 92,156 | 11,943 |
| Median follow-up years (interquartile range) | 0.67 (1.02) | 0.62 (1.07) |
| Number of cases | 369 | 54 |
| Crude incidence rate^†^ (95%CI) | 4.00 (3.62-4.43) | 4.52 (3.46-5.90) |
|  |  |  |
| **Lower extremity amputation** |  |  |
| Total follow-up person-years | 92,299 | 11,963 |
| Median follow-up years (interquartile range) | 0.67 (1.03) | 0.63 (1.07) |
| Number of cases | 103 | 15 |
| Crude incidence rate^†^ (95%CI) | 1.12 (0.92-1.35) | 1.25 (0.76-2.08) |
|  |  |  |
| **Intention-to-treat approach** |  |  |
| **Hospitalization for critical limb ischemia** |  |  |
| Total follow-up person-years | 128,875 | 19,168 |
| Median follow-up years (interquartile range) | 1.23 (1.23) | 1.54 (1.23) |
| Number of cases | 555 | 104 |
| Crude incidence rate^†^ (95%CI) | 4.31 (3.96-4.68) | 5.43 (4.48-6.58) |
|  |  |  |
| **Lower extremity amputation** |  |  |
| Total follow-up person-years | 129,162 | 19,227 |
| Median follow-up years (interquartile range) | 1.23 (1.23) | 1.55 (1.23) |
| Number of cases | 170 | 29 |
| Crude incidence rate^†^ (95%CI) | 1.32 (1.13-1.53) | 1.51 (1.05-2.17) |
|  |  |  |

GLP-1RA, glucagon-like peptide 1 receptor agonist; SGLT-2i, sodium-glucose cotransporter type-2 inhibitor; CI, confidence interval.

^†^Crude incidence rate per 1,000 person-years

1. **After propensity score matching**

|  | 1:1 PS-matched cohort (N=26,756) | |
| --- | --- | --- |
|  | SGLT-2i initiators  (n=13,378) | GLP-1 RA initiators  (n=13,378) |
|  |  |  |
| **On-treatment approach** |  |  |
| **Hospitalization for critical limb ischemia** |  |  |
| Total follow-up person-years | 11,521 | 11,366 |
| Median follow-up years (interquartile range) | 0.69 (1.05) | 0.63 (1.08) |
| Number of cases | 56 | 49 |
| Incidence rate^†^ after PS matching (95%CI) | 4.86 (3.74-6.32) | 4.31 (3.26-5.70) |
|  |  |  |
| **Lower extremity amputation** |  |  |
| Total follow-up person-years | 11,544 | 11,385 |
| Median follow-up years (interquartile range) | 0.69 (1.05) | 0.64 (1.08) |
| Number of cases | 18 | 14 |
| Incidence rate^†^ after PS mactching(95%CI) | 1.56 (0.98-2.47) | 1.23 (0.73-2.08) |
|  |  |  |
| **Intention-to-treat approach** |  |  |
| **Hospitalization for critical limb ischemia** |  |  |
| Total follow-up person-years | 17,002 | 18,218 |
| Median follow-up years (interquartile range) | 1.37 (1.24) | 1.56 (1.23) |
| Number of cases | 92 | 91 |
| Incidence rate^†^ (95%CI) | 5.41 (4.41-6.64) | 5.00 (4.07-6.13) |
|  |  |  |
| **Lower extremity amputation** |  |  |
| Total follow-up person-years | 17,038 | 18,272 |
| Median follow-up years (interquartile range) | 1.37 (1.24) | 1.56 (1.22) |
| Number of cases | 39 | 26 |
| Incidence rate^†^ after PS matching (95%CI) | 2.29 (1.67-3.13) | 1.42 (0.97-2.09) |
|  |  |  |

GLP-1RA, glucagon-like peptide 1 receptor agonist; SGLT-2i, sodium-glucose cotransporter type-2 inhibitor; CI, confidence interval.

^†^Incidence rate rate per 1,000 person-years

Supplementary Table 8 Auxiliary analyses: Hazard ratios of hospitalization for critical limb ischemia and lower extremity amputation comparing SGLT-2i initiators versus GLP-1 RA initiators after excluding patients with peripheral vascular disease or critical limb ischemia or lower extremity amputation at baseline - After propensity score matching

|  | **SGLT-2i initiators versus GLP-1 RA initiators** | | |
| --- | --- | --- | --- |
|  | GLP-1RA  (n= 13,009) | SGLT-2i  (n=13,009) | |
|  |  | Hazard ratio (95%CI) | |
|  |  | On-treatment approach | Intention-to-treat approach |
| **Hospitalization for critical limb ischemia** | Reference | 1.10 (0.67-1.82) | 1.23 (0.83-1.83) |
| **Lower extremity amputation** | Reference | 1.16 (0.50-2.68) | 1.67 (0.89-3.14) |

DPP-4i, dipeptidyl peptidase-4 inhibitor; GLP-1RA, glucagon-like peptide 1 receptor agonist; SGLT-2i, sodium-glucose cotransporter type-2 inhibitor; CI, confidence interval.

Supplementary Table 9 Auxiliary analyses: Hazard ratios of hospitalization for critical limb ischemia and lower extremity amputation comparing empagliflozin and dapagliflozin initiators versus glucagon-like peptide 1 receptor agonist (GLP-1 RA) initiators - After propensity score matching. (On-treatment approach)

|  | **SGLT-2i initiators versus GLP-1 RA initiators** | | |
| --- | --- | --- | --- |
|  | GLP-1RA | **Empagliflozin**  (n=12,211) | **Dapagliflozin**  (n=12,173) |
|  |  | Hazard ratio  (95%CI) | Hazard ratio  (95%CI) |
| **Hospitalization for critical limb ischemia** | Reference | 1.15 (0.75-1.74) | 1.26 (0.83-1.91) |
| **Lower extremity amputation** | Reference | 1.16 (0.55-2.44) | 1.31 (0.62-2.76) |

DPP-4i, dipeptidyl peptidase-4 inhibitor; GLP-1RA, glucagon-like peptide 1 receptor agonist; SGLT-2i, sodium-glucose cotransporter type-2 inhibitor; CI, confidence interval.

Supplementary Table 10 Demographics, smoking, clinical parameters, comorbidities, other medication use, and healthcare utilization at baseline among initiators of sodium glucose cotransporter 2 inhibitors (SGLT2i) and dipeptidyl peptidase-4 inhibitor (DPP-4i) before and after PS matching

|  | Before matching (n=589,327) | | | 1:1 PS-matched cohort (n=56,516) | | |
| --- | --- | --- | --- | --- | --- | --- |
| **Covariates** | SGLT2i initiators  ( n=32,469) | DPP-4i initiators  initiators  ( n=556,858) | Standardized difference | SGLT2i  initiators  ( n=28,258) | DPP-4i initiators  ( n=28,258) | Standardized difference |
| **Demographics** | | | | | | |
| Age in years, mean (SD) | 55.13 (12.66) | 62.45 (13.86) | -0.552 | 54.74 (12.57) | 54.65 (12.83) | 0.007 |
| Men | 58.78 | 53.96 | 0.097 | 58.78 | 58.46 | 0.006 |
| Overweight and obesity, % | 4.68 | 1.74 | 0.167 | 4.80 | 4.66 | 0.007 |
| Smoking | 1.85 | 1.38 | 0.037 | 1.89 | 2.03 | -0.011 |
| **Clinical parameters** | | | | | | |
| HbA1c, % |  |  |  |  |  |  |
| >9.0 | 23.17 | 16.49 | 0.168 | 22.74 | 22.84 | -0.003 |
| 7.0-9.0 | 43.56 | 25.89 | 0.378 | 44.08 | 44.25 | -0.003 |
| <7.0 | 12.53 | 11.23 | 0.040 | 12.26 | 12.20 | 0.002 |
| Missing | 20.74 | 46.40 | -0.565 | 20.92 | 20.71 | 0.005 |
| Mean (SD)^b^ | 8.49 (1.79) | 8.54 (2.08) | -0.028 | 8.48 (1.78) | 8.56 (1.93) | -0.045 |
| eGFR, mL/min |  |  |  |  |  |  |
| ≥90 | 41.65 | 20.33 | 0.474 | 42.52 | 42.41 | 0.002 |
| 60-89 | 29.96 | 20.37 | 0.222 | 29.50 | 29.73 | -0.005 |
| 30-59 | 7.82 | 12.89 | -0.167 | 7.09 | 6.96 | 0.005 |
| <30 | 0.49 | 4.63 | -0.264 | 0.31 | 0.35 | -0.006 |
| Missing | 20.08 | 41.78 | -0.483 | 20.57 | 20.55 | 0.001 |
| Mean (SD)^b^ | 88.45 (20.95) | 74.69 (28.04) | 0.556 | 89.30 (20.46) | 89.19 (21.42) | 0.005 |
| LDL-cholesterol, mg/dL |  |  |  |  |  |  |
| >140 | 9.08 | 6.69 | 0.089 | 8.99 | 9.11 | -0.004 |
| 120-140 | 9.85 | 6.64 | 0.117 | 9.71 | 9.72 | <-0.001 |
| 100-119 | 15.04 | 9.55 | 0.168 | 15.03 | 15.14 | -0.003 |
| <100 | 39.14 | 23.84 | 0.334 | 39.15 | 38.99 | 0.003 |
| Missing | 26.88 | 53.28 | -0.559 | 27.12 | 27.04 | 0.002 |
| Mean (SD)^b^ | 101.41 (33.19) | 103.41 (34.80) | -0.059 | 101.26 (33.05) | 101.75 (33.60) | -0.015 |
| SBP, mmHg |  |  |  |  |  |  |
| >160 | 2.96 | 3.26 | -0.018 | 2.98 | 2.79 | 0.011 |
| 140-160 | 12.70 | 12.20 | 0.015 | 12.95 | 12.91 | 0.001 |
| 120-139 | 27.74 | 24.36 | 0.077 | 28.48 | 28.23 | 0.006 |
| <120 | 8.96 | 8.51 | 0.016 | 9.19 | 9.11 | 0.003 |
| Missing | 47.63 | 51.67 | -0.081 | 46.40 | 46.96 | -0.011 |
| Mean (SD)^b^ | 132.86 (16.34) | 133.41 (17.23) | -0.033 | 132.78 (16.20) | 132.63 (16.19) | 0.009 |
| **Comorbidities** | | | | | | |
| Hypertension | 64.14 | 70.19 | -0.129 | 63.93 | 64.09 | -0.003 |
| Ischemic heart disease | 20.96 | 22.25 | -0.031 | 20.30 | 20.39 | -0.002 |
| Myocardial infarction | 2.82 | 2.81 | 0.001 | 2.79 | 2.87 | -0.005 |
| Coronary artery angioplasty or stenting | 2.65 | 3.10 | -0.027 | 2.62 | 2.63 | <-0.001 |
| CABG | 0.73 | 1.29 | -0.056 | 0.73 | 0.73 | 0.001 |
| Cerebrovascular disease | 7.81 | 17.57 | -0.296 | 7.49 | 7.36 | 0.005 |
| Ischemic stroke | 4.78 | 11.18 | -0.238 | 4.60 | 4.53 | 0.003 |
| Hemorrhagic stroke | 1.33 | 2.65 | -0.095 | 1.33 | 1.49 | -0.014 |
| Cardiac dysrhythmia | 6.49 | 9.02 | -0.095 | 6.40 | 6.38 | 0.001 |
| Congestive heart failure | 6.78 | 9.97 | -0.115 | 6.24 | 6.51 | -0.011 |
| Peripheral vascular disease | 2.01 | 4.22 | -0.127 | 1.96 | 1.90 | 0.004 |
| Hyperlipidemia | 75.02 | 65.21 | 0.215 | 75.59 | 75.55 | 0.001 |
| Chronic kidney disease | 6.64 | 12.03 | -0.186 | 6.45 | 6.31 | 0.006 |
| Charlson comorbidity index, mean (SD) | 2.29 (1.61) | 2.66 (1.81) | -0.220 | 2.27 (1.60) | 2.28 (1.59) | -0.006 |
| **Anti-hyperglycemic medication use** | | | | | | |
| Any insulin | 19.55 | 29.96 | -0.243 | 18.42 | 18.10 | 0.008 |
| Basal insulin | 9.54 | 10.28 | -0.025 | 9.05 | 8.84 | 0.007 |
| Premixed insulin | 6.61 | 5.35 | 0.053 | 5.78 | 5.76 | 0.001 |
| Metformin | 87.23 | 87.27 | -0.001 | 99.79 | 99.84 | -0.012 |
| Sulfonylurea | 45.46 | 57.11 | -0.235 | 49.04 | 48.93 | 0.002 |
| Glinides | 0.83 | 1.53 | -0.065 | 0.87 | 0.82 | 0.006 |
| Pioglitazone | 15.27 | 10.51 | 0.142 | 15.63 | 15.51 | 0.004 |
| α-glucosidase inhibitors | 10.78 | 13.77 | -0.091 | 10.26 | 10.15 | 0.004 |
| GLP1-RA | 1.16 | 0.15 | 0.126 | 0.89 | 0.80 | 0.010 |
| Number of oral anti-hyperglycemic medications^c^, mean (SD) | 1.61 (0.88) | 2.23 (0.96) | -0.676 | 1.76 (0.77) | 1.76 (0.79) | 0.006 |
| **Non- anti-hyperglycemic medication use** | | | | | | |
| ACEIs or ARBs | 57.29 | 58.09 | -0.016 | 57.03 | 56.91 | 0.002 |
| β blockers | 33.54 | 34.77 | -0.026 | 33.02 | 33.11 | -0.002 |
| Calcium channel blockers | 28.50 | 39.68 | -0.237 | 28.52 | 28.63 | -0.002 |
| Diuretics | 13.24 | 22.99 | -0.255 | 12.69 | 12.53 | 0.005 |
| Other anti-hypertensive agents | 4.66 | 7.66 | -0.125 | 4.57 | 4.66 | -0.004 |
| Nitrates | 11.81 | 14.66 | -0.084 | 11.47 | 11.47 | <-0.001 |
| Ivabradine | 0.26 | 0.08 | 0.045 | 0.19 | 0.21 | -0.003 |
| Valsartan+sacubitril | 0.34 | 0.05 | 0.066 | 0.25 | 0.25 | 0.000 |
| Aldactone | 3.71 | 4.90 | -0.059 | 3.46 | 3.38 | 0.005 |
| Eplerenone | 0.07 | 0.03 | 0.018 | 0.05 | 0.07 | -0.007 |
| Anti-arrhythmic agents | 2.90 | 5.24 | -0.119 | 2.82 | 2.75 | 0.005 |
| Digoxin | 1.31 | 2.51 | -0.088 | 1.21 | 1.19 | 0.002 |
| Aspirin | 28.21 | 35.41 | -0.155 | 27.88 | 27.96 | -0.002 |
| Clopidogrel | 6.13 | 8.27 | -0.083 | 5.76 | 5.73 | 0.001 |
| Warfarin | 0.74 | 1.72 | -0.089 | 0.69 | 0.64 | 0.006 |
| New oral anticoagulant | 1.82 | 1.59 | 0.018 | 1.73 | 1.78 | -0.003 |
| Statins | 61.44 | 50.47 | 0.222 | 62.23 | 61.94 | 0.006 |
| Fibrates | 13.04 | 11.31 | 0.053 | 13.24 | 13.29 | -0.002 |
| Number of cardiovascular-related medications ^d^, mean (SD) | 2.65 (1.87) | 2.94 (2.07) | -0.151 | 2.63 (1.86) | 2.63 (1.88) | 0.002 |
| **Healthcare utilization** | | | | | | |
| Echocardiography | 11.58 | 17.75 | -0.175 | 11.43 | 11.64 | -0.007 |
| Carotid ultrasonography | 3.47 | 7.50 | -0.177 | 3.42 | 3.43 | -0.001 |
| Transcranial ultrasonography % | 2.22 | 4.94 | -0.147 | 2.19 | 2.23 | -0.003 |
| Lower extremity arterial ultrasonography | 0.80 | 1.22 | -0.042 | 0.80 | 0.78 | 0.003 |
| 24-hour ECG examination | 2.63 | 3.46 | -0.048 | 2.59 | 2.72 | -0.008 |
| BNP, proBNP, or NT-proBNP test | 4.20 | 8.13 | -0.164 | 3.95 | 4.01 | -0.003 |
| Prescriber’s specialty |  |  |  |  |  |  |
| Cardiologist or cardiovascular surgeon | 22.84 | 16.88 | 0.150 | 21.85 | 21.82 | 0.001 |
| Endocrinologist | 33.37 | 27.31 | 0.132 | 33.94 | 33.46 | 0.010 |
| Other specialty | 43.79 | 55.81 | -0.242 | 44.21 | 44.72 | -0.010 |
| Number of hospitalizations, mean (SD) | 0.18 (0.48) | 0.54 (0.72) | -0.582 | 0.18 (0.48) | 0.18 (0.42) | 0.004 |
| Number of hospitalization due to CV-related episodes^f^, mean (SD) | 0.14 (0.42) | 0.41 (0.66) | -0.502 | 0.14 (0.42) | 0.13 (0.37) | 0.004 |
| Number of hospitalization due to genito-urinary infection-related episodes^g^, mean (SD) | 0.02 (0.15) | 0.05 (0.26) | -0.174 | 0.02 (0.15) | 0.02 (0.13) | 0.009 |
| Number of hospitalization due to diabetic ketoacidosis, mean (SD) | 0.00 (0.04) | 0.01 (0.08) | -0.057 | 0.00 (0.04) | 0.00 (0.04) | -0.001 |
| Number of outpatient visits, mean (SD) | 16.47 (10.46) | 18.33 (12.21) | -0.164 | 16.41 (10.41) | 16.50 (10.93) | -0.009 |
| Number of outpatient visits due to CV-related episodes^f^, mean (SD) | 6.63 (4.71) | 6.73 (5.57) | -0.021 | 6.57 (4.67) | 6.57 (5.00) | <-0.001 |
| Number of outpatient visits due to genito-urinary infection-related episodes ^g^, mean (SD) | 0.33 (1.32) | 0.32 (1.29) | 0.009 | 0.33 (1.32) | 0.33 (1.41) | -0.001 |

c statistis for PS model: 0.901

DPP-4i, dipeptidyl peptidase-4 inhibitor; ACEIs, angiotensin-converting enzyme inhibitors; ARBs, angiotensin Ⅱ receptor blockers; BNP, B-type natriuretic peptide; CABG, coronary artery bypass graft surgery; CV, cardiovascular;ECG, electrocardiogram; eGFR, estimated glomerular filtration rate; GLP-1RAs, glucagon-like peptide-1 receptor agonists; HbA1c, glycated hemoglobin; LDL, low-density lipoprotein; NT, N-terminal; PPI, proton pump inhibitors; PS, propensity score; SBP, systolic blood pressure; SD, standard deviation; SGLT2is, sodium glucose cotransporter 2 inhibitors.

^a^Data are presented as proportion of patents unless otherwise specified.

^b^Among patients without missing value.

^c^Oral anti-hyperglycemic medications included metformin, sulfonylureas. glinides, pioglitazone, α-glucosidase inhibitors, and dipeptidyl peptidase-4 inhibitors.

^d^CV-related medications included ACEIs or ARBs, β blockers, calcium channel blockers, diuretics, other anti-hypertensive agents, nitrates, anti-arrhythmic agents, digoxin, aspirin, clopidogrel, warfarin, new oral anti-coagulants, statins, and fibrates.

^e^The specialty of the physicians who prescribed the study drugs.

^f^Cardiovascular-related episodes included hypertension, ischemic heart disease, myocardial infarction, congestive heart failure, cerebrovascular disease, peripheral vascular disease, and hyperlipidemia.

.^g^Genito-urinary infection-related-episodes included urinary tract infection, genital tract infection, fournier gangrene, and candidiasis.

Supplementary Table 11 Follow-up duration, number of cases, and incidence of critical limb ischemia and lower extrimity amputation among sodium-glucose cotransporter type-2 inhibitor (SGLT-2i) and dipeptidyl peptidase-4 inhibitor (DPP-4i) initiators (a) before and (b) after propensity score matching.

1. Before propensity score matching

|  | SGLT-2i initiators  ( N=32,469) | DPP-4i initiators  ( N=556,858) |
| --- | --- | --- |
|  |  |  |
| **On-treatment approach** |  |  |
| **Hospitalization for critical limb ischemia** |  |  |
| Total follow-up person-years | 26,180 | 606,586 |
| Median follow-up years (interquartile range) | 0.64 (0.97) | 1.00 (1.66) |
| Number of cases | 75 | 3,854 |
| Crude incidence rate^†^ (95%CI) | 2.86 (2.28-3.59) | 6.35 (6.16-6.56) |
|  |  |  |
| **Lower extremity amputation** |  |  |
| Total follow-up person-years | 26,200 | 608,591 |
| Median follow-up years (interquartile range) | 0.64 (0.97) | 1.00 (1.66) |
| Number of cases | 19 | 1,124 |
| Crude incidence rate^†^ (95%CI) | 0.73 (0.46-1.14) | 1.85 (1.74-1.96) |
|  |  |  |
| **Intention-to-treat approach** |  |  |
| **Hospitalization for critical limb ischemia** |  |  |
| Total follow-up person-years | 37,179 | 912,816 |
| Median follow-up years (interquartile range) | 1.16 (1.14) | 2.00 (0.62) |
| Number of cases | 114 | 6,183 |
| Crude incidence rate^†^ (95%CI) | 3.07 (2.55-3.68) | 6.77 (6.61-6.94) |
|  |  |  |
| **Lower extremity amputation** |  |  |
| Total follow-up person-years | 37,236 | 916,710 |
| Median follow-up years (interquartile range) | 1.16 (1.14) | 2.00 (0.61) |
| Number of cases | 30 | 2,001 |
| Crude incidence rate^†^ (95%CI) | 0.81 (0.56-1.15) | 2.18 (2.09-2.28) |

DPP-4i, dipeptidyl peptidase-4 inhibitor; SGLT-2i, sodium-glucose cotransporter type-2 inhibitor; CI, confidence interval.

^†^Crude incidence rate rate per 1,000 person-years

1. After propensity score matching

|  | 1:1 PS-matched cohort  (N=56,516) | |
| --- | --- | --- |
|  | SGLT-2i initiators  ( N= 28,258) | DPP-4i initiators  ( N=28,258) |
|  |  |  |
| **On-treatment approach** |  |  |
| **Hospitalization for critical limb ischemia** |  |  |
| Total follow-up person-years | 22,839 | 30,266 |
| Median follow-up years (interquartile range) | 0.63 (0.98) | 0.96 (1.63) |
| Number of cases | 60 | 71 |
| Incidence rate^†^ after PS matching (95%CI) | 2.63 (2.04-3.38) | 2.35 (1.86-2.96) |
|  |  |  |
| **Lower extremity amputation** |  |  |
| Total follow-up person-years | 22,854 | 30,303 |
| Median follow-up years (interquartile range) | 0.63 (0.98) | 0.96 (1.63) |
| Number of cases | 15 | 24 |
| Incidence rate^†^ after PS mactching(95%CI) | 0.66 (0.40-1.09) | 0.79 (0.53-1.18) |
|  |  |  |
| **Intention-to-treat approach** |  |  |
| **Hospitalization for critical limb ischemia** |  |  |
| Total follow-up person-years | 32,355 | 44,148 |
| Median follow-up years (interquartile range) | 1.16 (1.17) | 2.00 (0.85) |
| Number of cases | 91 | 111 |
| Incidence rate^†^ after PS matching (95%CI) | 2.81 (2.29-3.45) | 2.51 (2.09-3.03) |
|  |  |  |
| **Lower extremity amputation** |  |  |
| Total follow-up person-years | 32,404 | 44,215 |
| Median follow-up years (interquartile range) | 1.16 (1.17) | 2.00 (0.85) |
| Number of cases | 22 | 41 |
| Incidence rate^†^ after PS matching (95%CI) | 0.68 (0.45-1.03) | 0.93 (0.68-1.26) |

DPP-4i, dipeptidyl peptidase-4 inhibitor; SGLT-2i, sodium-glucose cotransporter type-2 inhibitor; CI, confidence interval.

^†^Incidence rate per 1,000 person-years

Supplementary Table 12 Hazard ratios of hospitalization for critical limb ischemia and lower extrimity amputation comparing sodium-glucose cotransporter type-2 inhibitor (SGLT-2i) initiators versus dipeptidyl peptidase-4 inhibitor (DPP-4i) initiators. (N = 589,327)

|  | DPP-4i | SGLT-2i | |
| --- | --- | --- | --- |
|  |  | On-treatment approach | Intention-to-treat approach |
|  |  | Hazard ratio  (95%CI) | Hazard ratio  (95%CI) |
| **Hospitalization for critical limb ischemia** |  |  |  |
| Crude | Reference | 0.41 (0.33-0.52) | 0.43 (0.35-0.51) |
| After PS matching | Reference | 1.06 (0.75-1.50) | 1.08 (0.82-1.43) |
|  |  |  |  |
| **Lower extremity amputation** |  |  |  |
| Crude | Reference | 0.35 (0.22-0.56) | 0.35 (0.24-0.50) |
| After PS matching | Reference | 0.80 (0.42-1.53) | 0.73 (0.43-1.23) |

DPP-4i, dipeptidyl peptidase-4 inhibitor; SGLT-2i, sodium-glucose cotransporter type-2 inhibitor; CI, confidence interval; PS, propensity score.

Supplementary Table 13 Subgroup analyses: hazard ratios of hospitalization for critical limb ischemia and lower extrimity amputation comparing sodium-glucose cotransporter type-2 inhibitor (SGLT-2i) initiators versus dipeptidyl peptidase-4 inhibitor (DPP-4i) initiators stratified by age, with or without cardiovascular disease, and chronic kidey disease.

|  |  | DPP-4i | SGLT-2i |
| --- | --- | --- | --- |
|  | N= |  | Hazard ratio  (95%CI) |
| **Hospitalization for critical limb ischemia** |  |  |  |
| **Age(years)** |  |  |  |
| ≤60 | 37,428 | Reference | 1.11 (0.65-1.90) |
| >60 | 19,098 | Reference | 1.04 (0.69-1.56) |
| **Cadiovascular diseases** |  |  |  |
| Yes | 18,392 | Reference | 1.24 (0.85-1.80) |
| No | 38,084 | Reference | 0.87 (0.45-1.71) |
| **Chronic kidney disease** |  |  |  |
| Yes or eGFR<60 (mL/min/1.73m^2^) | 6,922 | Reference | 1.22 (0.73-2.06) |
| No or eGFR≥60 | 49,552 | Reference | 0.80 (0.54-1.19) |
|  |  |  |  |
| **Lower extremity amputation** |  |  |  |
| **Age(years)** |  |  |  |
| ≤60 | 37,428 | Reference | 0.98 (0.42-2.27) |
| >60 | 19,098 | Reference | 0.64 (0.25-1.60) |
| **Cadiovascular diseases** |  |  |  |
| Yes | 18,392 | Reference | 2.06 (0.81-5.25) |
| No | 38,084 | Reference | 1.01 (0.31-3.22) |
| **Chronic kidney disease** |  |  |  |
| Yes or eGFR<60 (mL/min/1.73m^2^) | 6,922 | Reference | 0.69 (0.25-1.91) |
| No or eGFR≥60 | 49,552 | Reference | 0.78 (0.36-1.67) |

DPP-4i, dipeptidyl peptidase-4 inhibitor; SGLT-2i, sodium-glucose cotransporter type-2 inhibitor; CI, confidence interval; eGFR, estimated glomerular filtration rate.

Supplementary Figure 1 Study cohort assembly (SGLT-2i versus DPP-4i).

| Adults with diabetes mellitus identified from the NHIRD between Jan 1 2012 and December 31 2017 (N= 2,026,617) | |  |  |
| --- | --- | --- | --- |
|  |  |  |  |
|  |  | Excluded due to   - Patients with type 1 diabetes (N=82,060) | |
|  |  |  |  |
|  |  |  |  |
| Patients with type 2 diabetes (N= 1,944,557) | |  |  |
|  |  |  |  |
|  |  | Excluded due to   - Patients with no prescription of DPP-4i or SGLT-2i during study period (N= 1,115,818) | |
|  |  |  |  |
|  |  |  |  |
| Potential study population ( N= 828,739)   - Initiators of DPP-4i (N= 786,845) - Initiators of SGLT-2i (N= 41,368) - Initiators both of above (N= 526) | |  |  |
|  |  |  |  |
|  |  | Excluded due to   - Patients who received both DPP-4i and SGLT-2i on the index date (N = 526) - Patients who received more than one DPP-4i or more than one SGLT-2i on the index date (N = 90,424) - Patients who had ever used SGLT-2i therapy within two years before the index date for initiators of DPP-4i (N=1,058) - Patients who had ever used DPP-4i therapy within two years before the index date for initiators of SGLT-2i (N=3,289) | |
|  |  |  |  |
|  |  |  |  |
| - Eligible study population ( N=733,442) - Initiators of DPP-4i (N=695,371)   - Sitagliptin (N=260,233)  - Vildagliptin (N=179,971)  - Saxagliptin (N = 91,895)  - Linagliptin (N = 160,781)  - Alogliptin (N = 2,491)   - Initiators of SGLT-2i (N=38,071)   - Empagliflozin (N=16,026)  - Dapagliflozin (N=22,045) | |  |  |
|  |  |  |  |
|  |  | Excluded due to   - Age <20 years or age >100 years (N=1,343) - Patients who already had outpatient or inpatient diagnosis of the following diseases before study beginning: Organ transplantation (N=1,592);Cancer (N=94,188); Liver cirrhosis (N=17,920); Dialysis (N=20,803) - Those without at least 365 days of continuous enrollment before the index date (N=1,511) - Patients who were censored on the index date (N=6,067) - Patients with outcome occurrence on the index date (N=691) | |
|  |  |  |  |
|  |  |  |  |
| - Eligible study population ( N=589,327) - Initiators of DPP-4i (N=556,858)   - Sitagliptin (N=212,902)  - Vildagliptin (N=148,205)  - Saxagliptin (N = 75,398)  - Linagliptin (N = 118,405)  - Alogliptin (N = 1,948)   - Initiators of SGLT-2i (N=32,469)   - Empagliflozin (N=13,517)  - Dapagliflozin (N=18,952) | |  |  |

NHIRD, The Taiwan National Health Insurance Database ; DPP-4i, dipeptidyl peptidase-4 inhibitor; GLP-1RA, glucagon-like peptide 1 receptor agonist; SGLT-2i, sodium-glucose cotransporter type-2 inhibitor; eGFR, estimated glomerular filtration rate.

Supplementary Figure 2 Cumulative incidence curves of hospitalized critical limb ischemia and lower extremity amputation among diabetes patients initiating empagliflozin and those initiating dapagliflozin versus glucagon-like peptide 1 receptor agonist (GLP-1RA) use after propensity score matching.

| - Empagliflozin | |
| --- | --- |
| (a1) Hospitalized critical limb ischemia (vs GLP-1RA)  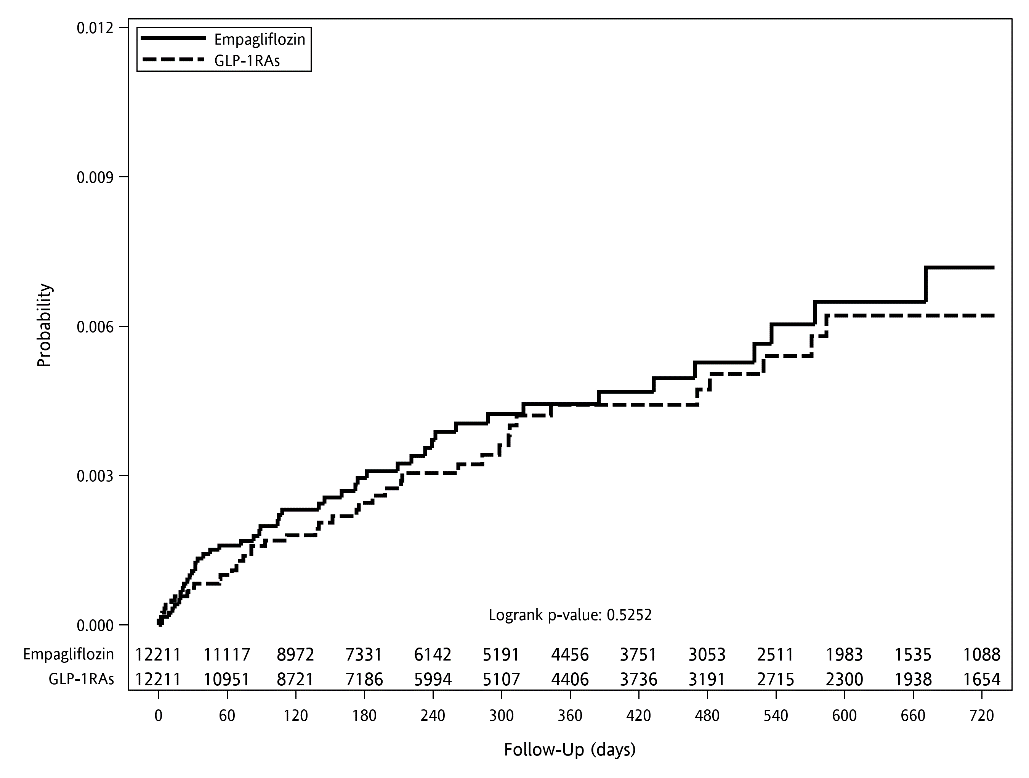 | **(b1) Lower extrimity amputation (vs GLP-1RA)**  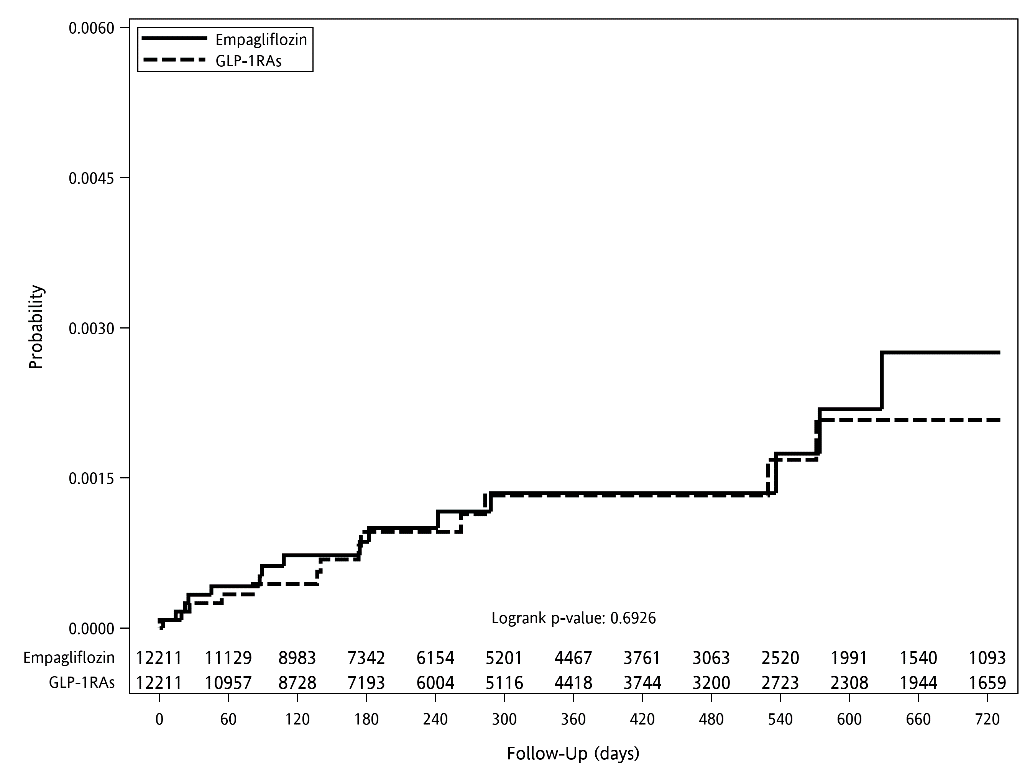 |
|  |  |
| - Dapagliflozin | |
| (a2) Hospitalized critical limb ischemia (vs GLP-1RA)  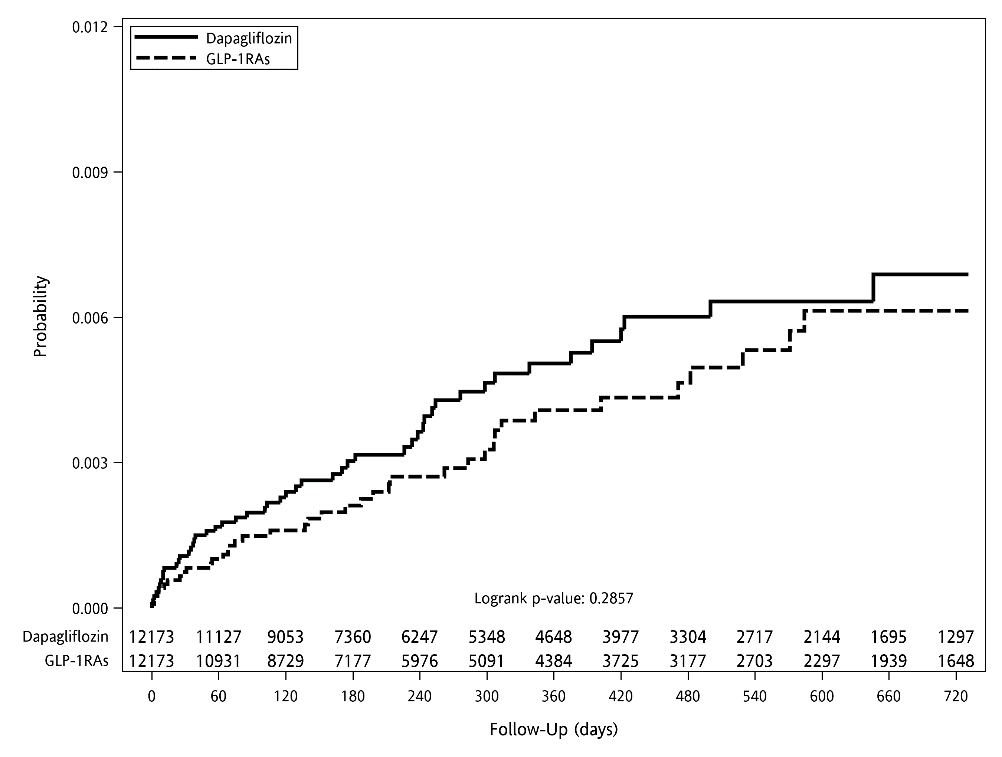 | **(b2) Lower extrimity amputation (vs GLP-1RA)**  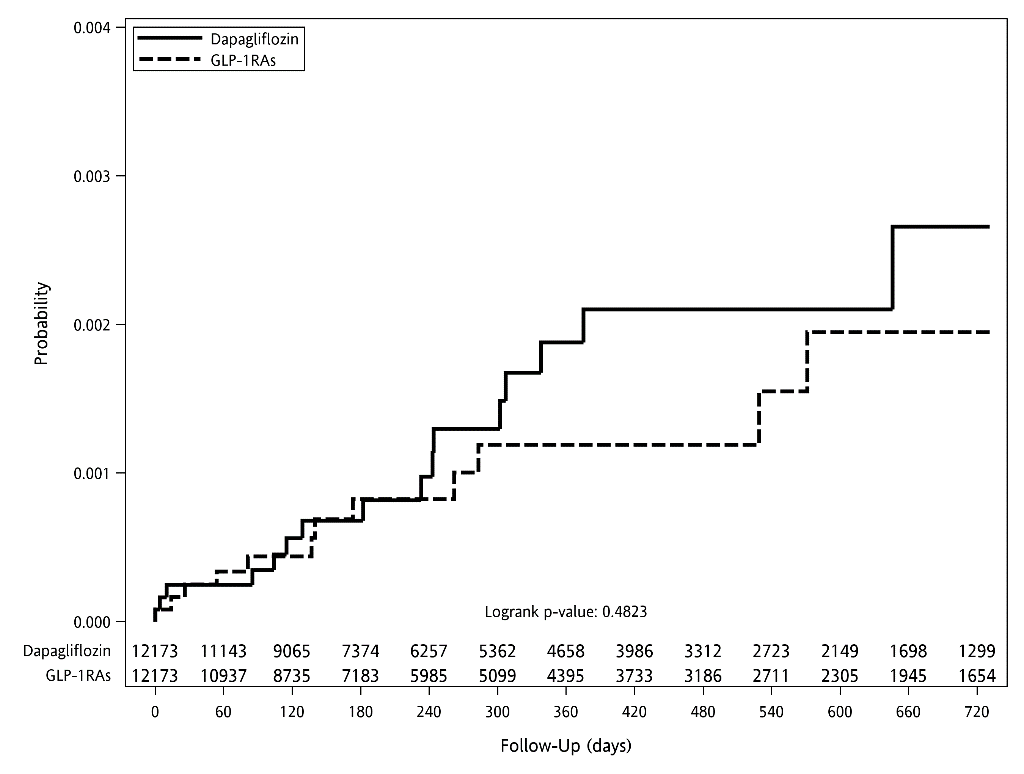 |

Supplementary Figure 3 Cumulative incidence curves of (a) hospitalized critical limb ischemia and (b) lower extremity amputation among diabetes patients initiating sodium-glucose cotransporter type-2 inhibitors (SGLT-2i) and dipeptidyl peptidase-4 inhibitors (DPP-4i) after propensity score matching. (On treatment approach: a1 and b1; Intention-to-treat approach: a2 and b2)

| (a1) Hospitalized critical limb ischemia (on treatment apprach)  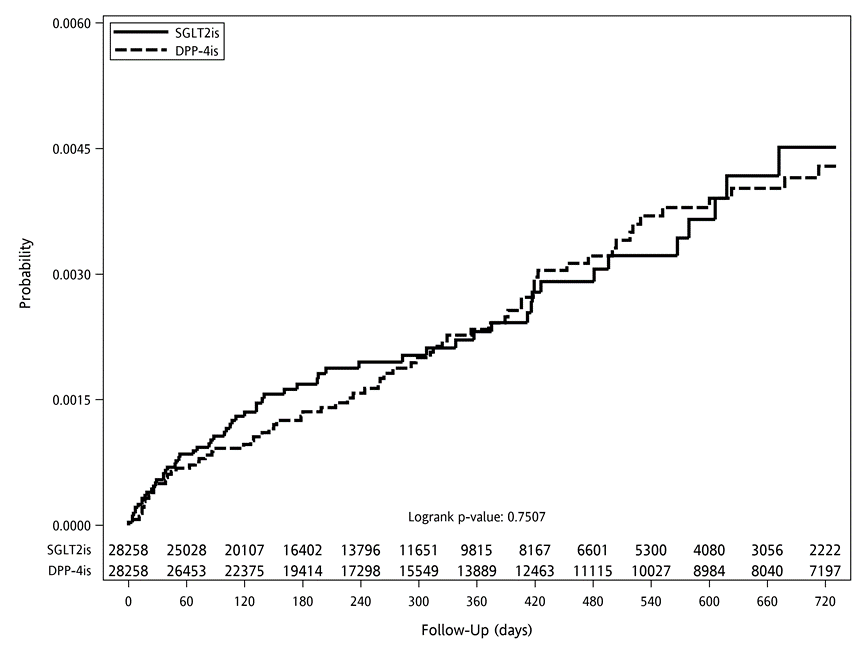 | (b1) Lower extrimity amputation (on treatment approach)  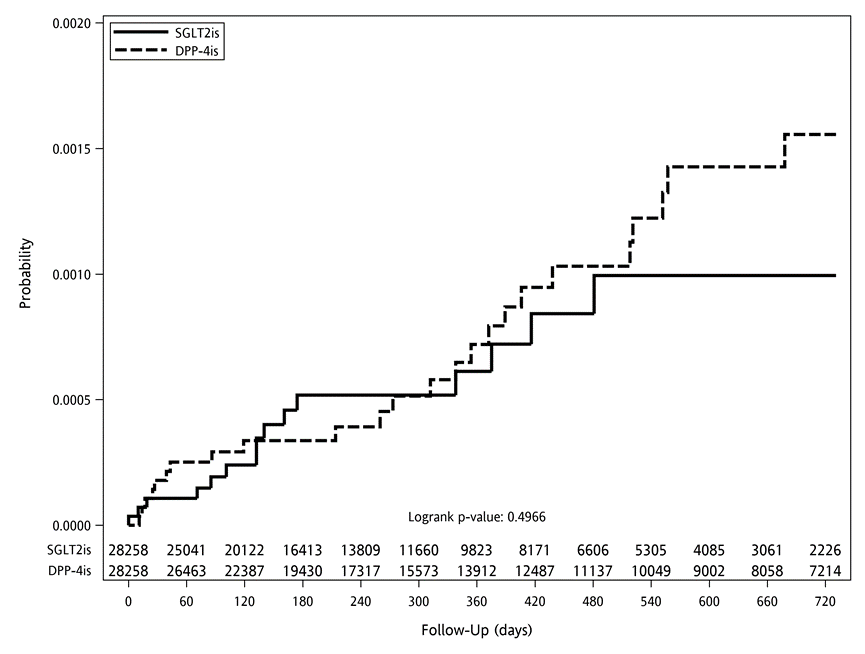 |
| --- | --- |
| (a2) Hospitalized critical limb ischemia (intention-to-treat approach)  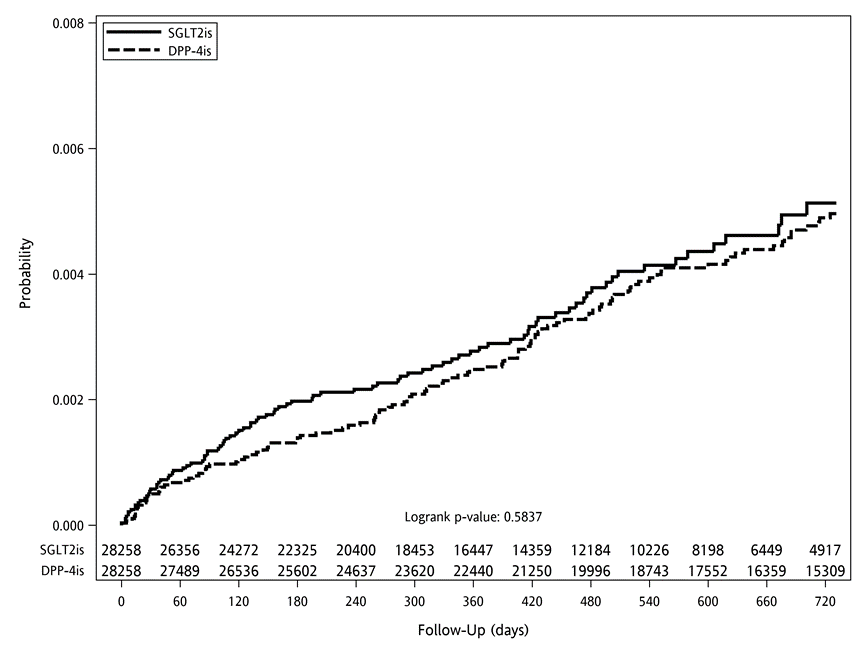 | **(b2) Lower extrimity amputation (intention-to-treat approach)**  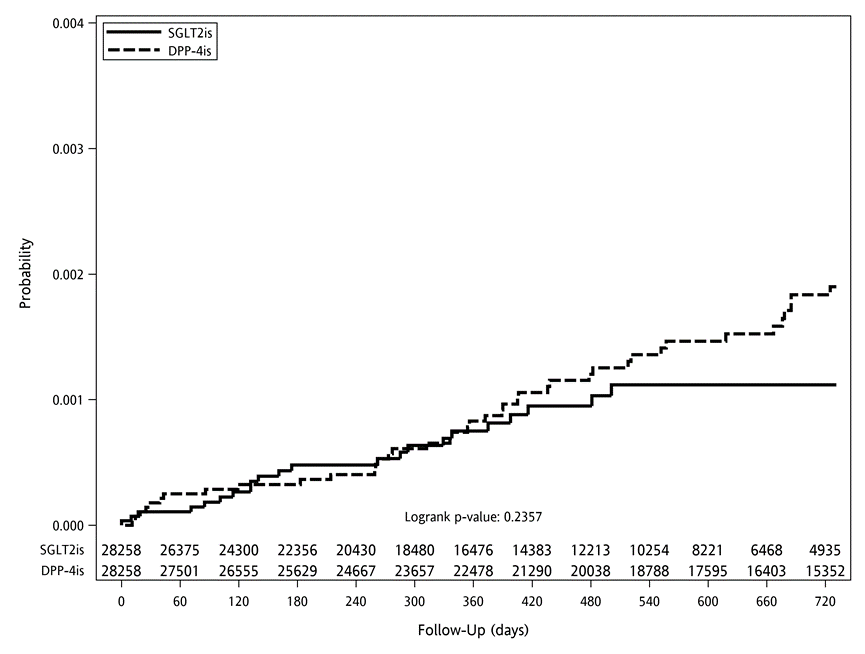 |
